# Performance evolution in Olympic swimming over the last 100 years: A retrospective stroke-based analysis

**DOI:** 10.1371/journal.pone.0361289

**Published:** 2026-10-26

**Authors:** Ayça Anameriç, Elif Cengizel, Burcu Ertaş Dölek, Hacı Ahmet Pekel

**Affiliations:** 1 Department of Coaching Education, Çanakkale Onsekiz Mart University, Çanakkale, Turkey; 2 Department of Coaching Education, Faculty of Sport Sciences, Gazi University, Turkey; 3 Department of Coaching Education, Faculty of Sport Sciences, Ankara University, Turkey; Universidade Estadual Paulista Julio de Mesquita Filho - Campus de Bauru, BRAZIL

## Abstract

This retrospective study examined the historical progression of Olympic swimming performance across 24 Olympic Games from Paris 1924 to Paris 2024. Finalist race data for 1553 women and 1641 men were extracted from the official World Aquatics database, including final times, reaction times, and calculated swimming speeds across all strokes (freestyle, backstroke, breaststroke, butterfly) and Olympic distances (50–1500 m). Across all strokes and distances, final times demonstrated substantial reductions over the century, with the most pronounced improvements observed in freestyle and long-distance events. The largest percentage improvements in performance were observed during specific Olympic cycles, including 1956, 1972, and 2008, which coincided with major developments in pool engineering, competition regulations, training methodologies, and swimsuit technology. Swimming speeds increased consistently in all events, while reaction-time analyses revealed significant improvements across Olympic Games during the period in which reaction-time data were recorded. Overall, these findings demonstrate substantial long-term improvements in Olympic swimming performance and provide a comprehensive historical overview of performance changes across strokes, distances, and sexes. The periods associated with the largest performance improvements coincided with major technological, biomechanical, and regulatory developments in the sport. The results may serve as valuable benchmarks for coaches, athletes, and sports scientists to evaluate long-term performance trends and prepare for future international competitions.

## Introduction

From past to present, the sport of swimming—like many other sports disciplines—has shown substantial progression and has evolved into a discipline widely practiced and embraced by large populations [1]. This discipline has been included in the Olympic program for more than a century, dating back to the first modern Olympic Games held in Athens in 1896; however, in the early 20th century, it was largely shaped and practiced by amateur athletes [2]. Among the four competitive stroke categories, freestyle is the fastest style swum in competition [3]. Analysis of swimming final times shows that breaststroke is the slowest of all techniques [2]. At the Olympic Games, breaststroke, butterfly, and backstroke events are contested over 100-m and 200-m for both genders, whereas freestyle events have been held over 50-m, 100-m, 200-m, 400-m, 800-m, and 1500-m for both genders since the 2020 Olympic Games [1]. Variations in distance and stroke lead to corresponding differences in physiological demands and biomechanical factors [4].

Swimming was incorporated into the Olympic program in 1896 with the first modern Olympic Games [5]. When examining the Olympic Games over time, numerous changes and developments become evident. Breaststroke is a discipline that evolved from other techniques and was formally separated from the butterfly stroke in the 1950s [6]. Until 1956, Olympic swimming competitions were held in three disciplines; at the Melbourne Olympic Games, the butterfly event was officially introduced into the Olympic program. An examination of the Olympic Games reveals variations over time in the number of participating female and male swimmers, the disciplines included, the race distances, and the performance outcomes [7]. A 15-meter underwater limit was introduced for starts and turns, and breaststroke swimmers were permitted a single dolphin kick during the underwater phase. Athletes’ performances continue to improve over time, leading to progressive changes in race results. One clear indicator of this advancement is the decreasing time gap between medalists and other finalists [2,8]. According to Dustker and Nayak [9], numerous parameters can contribute to athletic performance. To enhance swimmers’ performance, a wide range of technological developments has been used [10]. As the role of equipment and technology in sport continues to grow, the potential for both the sport and the athlete to improve increases accordingly [11]. Given that knowledge obtained under former competition regulations may not fully reflect the current competitive context, coaches should practice caution when integrating race-analysis information into modern training and performance strategies [12].

There are studies that have examined the historical development of swimming through analyses of World Championships and Olympic Games [2,13–15]. In addition, previous research has examined multiple aspects of the historical progression of Olympic swimming performance. For example, da Silva et al. [16] analyzed freestyle performance metrics—including reaction time, split times, speed, and final times—at the 2008–2016 Olympic Games, while Cengiz and Coşkun [17] investigated long-term performance trends across all strokes between 1896 and 2020. More recently, Veiga et al. [18] expanded this line of inquiry by modeling decade-long global performance trajectories using world-ranking data, demonstrating systematic improvements across strokes and distances despite seasonal fluctuations such as those induced by the COVID-19 pandemic. Although these studies have contributed substantially to our understanding of swimming performance evolution, they have generally focused on specific performance variables, limited Olympic periods, or world-ranking datasets. Consequently, there remains a need for research that comprehensively and systematically evaluates long-term changes in Olympic swimming performance across strokes, distances, reaction times, swimming speeds, and final race outcomes [19].

To the authors’ knowledge, no previous study has comprehensively examined Olympic swimming performance across a 100-year period while simultaneously evaluating all four competitive strokes (freestyle, backstroke, breaststroke, and butterfly), multiple race distances, final race times, reaction times, and swimming speeds within a single analytical framework. Previous investigations have generally focused on specific strokes, limited Olympic periods, selected performance variables, or world-ranking data rather than Olympic finalist performances. Therefore, the novelty of the present study lies not only in its century-long historical scope but also in its integrated stroke-based approach, enabling the identification of long-term performance changes and trends across all Olympic swimming disciplines. This comprehensive perspective provides a broader understanding of how swimming performance has evolved alongside technological, regulatory, and methodological developments throughout Olympic history. Therefore, this present study investigates the historical progression of Olympic swimming between 1924 and 2024, focusing on final times, reaction times, and swimming speeds in freestyle, backstroke, butterfly, and breaststroke events—disciplines in which performance continues to improve over time and fractions of a second remain decisive in competitive outcomes. The findings of this study are expected to provide valuable guidance for coaches and athletes preparing for upcoming national and international competitions.

## Method

### Study design and data source

The data for this retrospective study were obtained from publicly accessible competitive results on the official World Aquatics website and included 1553 female and 1641 male athletes who competed in the finals of the Olympic Games between 1924 and 2024. The dataset was accessed for research purposes on 03/08/2025. Although athlete names and performances were publicly available, the authors did not access or use any private or sensitive personal information, and all analyses were conducted using de-identified performance data. Swimming performance data from the final races of all Olympic years were included for each distance (50-m, 100-m, 200-m, 400-m, 800-m, 1500-m) across the four competitive strokes (freestyle, backstroke, butterfly, breaststroke). Ethical approval for the study was obtained from the Gazi University Ethics Commission (Research Code: 2022−1074).

### Performance variables

Swimming performance variables were categorized as final time (sec), reaction time (sec), and swimming speed (m·sec ^−^ ¹). Swimming speeds were calculated from the official World Aquatics final-time data by dividing the race distance by the corresponding completion time. Although swimming speed is mathematically derived from race time and distance, it was included to provide a standardized metric that facilitates comparisons across swimming events of different distances and strokes.

### Data processing

Mean finalist performances were used to represent each Olympic Games because Olympic finalists constitute the highest level of international swimming performance and provide a broader representation of elite competitive standards than individual winning performances. Furthermore, averaging finalist results reduces the influence of exceptional individual achievements and isolated performances, thereby providing a more stable estimate of competitive performance across Olympic cycles. For each Olympic Games between 1924 and 2024, mean and standard deviation values were calculated for all finalists’ performance variables. Percentage changes in swimming performance between consecutive Olympic Games were calculated by dividing the difference between two successive Games by the performance value of the earlier Games and multiplying the result by 100. Total percentage reduction was calculated by subtracting the mean final performance of the 2024 Olympic Games from the mean final performance of the first Olympic Games in which the stroke was contested, dividing this difference by the initial Olympic mean, and multiplying the result by 100.

### Data analysis

The Shapiro–Wilk test was used to assess the normality of data distribution. For comparisons of performance across Olympic Games, one-way analysis of variance was applied to normally distributed variables, whereas the Kruskal–Wallis test was used for non-normally distributed parameters. Post hoc pairwise comparisons were performed using Holm–Sidak, Tukey, or Dunn’s procedures. All statistical analyses were conducted using SigmaPlot 11.0 (Systat Software, Inc., San Jose, California, USA). The level of significance was set at 0.05.

## Results

### Freestyle – final time

Swimmers’ 50-m freestyle final times decreased significantly from the 1988 to the 2024 Olympic Games (25.76 ± .16 vs. 24.19 ± .24 sec in women, and 22.74 ± .34 vs. 21.52 ± .15 sec in men), with total reductions of 1.57 sec (6.1% difference) in women and 1.22 sec (5.4% difference) in men (Table 1). The largest percentage change in 50-m freestyle final time was recorded at the 2008 Olympic Games, with reductions of 2.4% in women and men. Percentage changes between consecutive Olympic Games for final performance times, swimming speeds, and reaction times are presented in S1–S9 Tables in S1 File.

**Table 1:** Final performance times of swimmers in the 50-, 100-, and 200-meter freestyle events at the Olympic Games (1924–2024) (sec).

	50-m		100-m		200-m	
	F	M	F	M	F	M
**1924**	NA	NA	75.12 ± 3.39	61.44 ± 1.49	NA	NA
**1928**	NA	NA	73.27 ± 1.71	60.46 ± 1.06	NA	NA
**1932**	NA	NA	68.80 ± 1.47	58.91 ± 0.48	NA	NA
**1936**	NA	NA	67.39 ± 1.09	58.64 ± 0.96	NA	NA
**1948**	NA	NA	67.90 ± 1.03	58.65 ± 1.06	NA	NA
**1952**	NA	NA	67.38 ± 0.61	58.29 ± 0.57	NA	NA
**1956**	NA	NA	64.63 ± 1.55	57.06 ± 1.00	NA	NA
**1960**	NA	NA	63.58 ± 1.31	55.93 ± 0.68	NA	NA
**1964**	NA	NA	61.48 ± 1.25	54.33 ± 0.87	NA	NA
**1968**	NA	NA	60.78 ± 0.59	53.36 ± 0.63	133.04 ± 2.11	118.46 ± 2.36
**1972**	NA	NA	59.45 ± 0.56	52.17 ± 0.61	126.76 ± 2.82	115.04 ± 1.69
**1976**	NA	NA	56.73 ± 0.50	51.43 ± 0.72	122.78 ± 2.09	111.38 ± 1.13
**1980**	NA	NA	56.78 ± 1.34	51.46 ± 0.60	121.92 ± 2.01	111.95 ± 1.19
**1984**	NA	NA	56.66 ± 0.74	50.45 ± 0.35	120.53 ± 1.27	110.54 ± 1.98
**1988**	25.76 ± 0.16	22.74 ± 0.34	55.78 ± 0.53	49.79 ± 0.66	119.68 ± 1.60	108.37 ± .63
**1992**	25.36 ± 0.34	22.39 ± 0.27	55.47 ± 0.67	49.59 ± 0.35	119.95 ± 1.43	108.19 ± 1.22
**1996**	25.37 ± 0.37	22.47 ± 0.24	55.37 ± 0.60	49.31 ± 0.43	119.95 ± 1.27	108.40 ± 0.50
**2000**	25.02 ± 0.48	22.19 ± 0.20^o^	54.76 ± 0.62^a-f^	48.95 ± 0.39^a-f^	118.92 ± .49^j^	107.43 ± 1.51^j^
**2004**	24.96 ± 0.19	22.11 ± 0.15^o,q^	54.58 ± 0.47^a-f^	48.80 ± 0.47^a-f^	118.69 ± 0.43^j^	106.49 ± 1.38^j,k^
**2008**	24.36 ± 0.28^o-q^	21.57 ± 0.14^o-s^	53.80 ± 0.50^a-h^	47.77 ± 0.40^a-i^	116.35 ± 1.34^j-m^	105.81 ± 1.48^j-m^
**2012**	24.45 ± 0.21^o^	21.68 ± 0.20^o-s^	53.50 ± 0.29^a-h^	47.87 ± 0.30^a-i^	116.26 ± 1.36^j-m^	105.60 ± 1.44^j-m^
**2016**	24.23 ± 0.22^o-q^	21.67 ± 0.23^o-s^	53.05 ± 0.25^a-j^	47.96 ± 0.25^a-i^	115.12 ± 0.91^j-n^	105.48 ± 0.44^j-m^
**2020**	24.23 ± 0.20^o-q^	21.60 ± 0.23^o-s^	52.61 ± 0.38^a-k^	47.64 ± 0.41^a-j^	115.01 ± 0.97^j-n^	104.87 ± 0.53^j-n^
**2024**	24.19 ± 0.24^o-q^	21.52 ± 0.15^o-s^	52.57 ± 0.32 ^a-k^	47.54 ± 0.50^a-j^	114.99 ± 1.07 ^j-n^	105.23 ± 0.55^j-n^

*Data are presented as mean*±SD. F: Female, M: Male, *NA: Not available. Each letter shown as a superscript denotes a significant difference with an Olympic Games. The letters respectively represent the following Olympics: a: 1924, b: 1928, c: 1932, d: 1936, e: 1948, f: 1952, g: 1956, h: 1960, i: 1964, j: 1968, k: 1972, l: 1976, m: 1980, n: 1984, o: 1988, p: 1992, q: 1996, r: 2000, s: 2004, t: 2008, u: 2012, v: 2016, w: 2020, x: 2024.*

The 100-m freestyle final time significantly decreased from the 1924 Olympic Games to the 2024 Olympic Games (75.12 ± 3.39 vs. 52.57 ± .32 sec in women, and 61.44 ± 1.49 vs. 47.54 ± .5 sec in men), corresponding to total reductions of 22.55 sec (30.0% difference) in women and 13.90 sec (22.6% difference) in men (Table 1). In the women’s 100-m freestyle, the largest percentage reductions in final times were observed at the 1932 (6.1%), 1956 (4.1%), and 1976 Olympic Games (4.6%). In men, the largest reductions were observed at the 1932 (2.6%), 1964 (2.9%), and 1972 Olympic Games (2.2%).

Swimmers’ 200-m freestyle final times showed a significant decrease from the 1968 Olympic Games to the 2024 Olympic Games (133.04 ± 2.11 vs. 114.99 ± 1.07 sec in women, and 118.46 ± 2.36 vs. 105.23 ± 0.55 sec in men), with total reductions of 18.05 sec (13.6% difference) in women and 13.23 sec (11.2% difference) in men (Table 1). The largest percentage reductions in 200-m freestyle final times were observed in women in 1972 (4.7%) and 2008 (2%), and in men in 1972 (2.9%) and 1976 (3.2%).

From the 1924 to the 2024 Olympic Games, 400-m freestyle final times decreased significantly (369.05 ± 10.95 vs. 241.34 ± 2.49 sec in women, and 314.88 ± 13.09 vs. 223.72 ± 2.03 sec in men), corresponding to total reductions of 127.71 sec (34.6% difference) in women and 91.16 sec (29.0% difference) in men (Table 2). The largest percentage reductions in 400-m final times were observed in women in 1936 (4.5%), 1960 (4.2%), and 1972 (5.9%), and in men in 1932 (5.4%), 1952 (4.3%), and 1972 (4.7%).

**Table 2:** Final performance times of swimmers in the 400-, 800-, and 1500-meter freestyle events at the Olympic Games (1924–2024) (sec).

	400-m		800-m		1500-m	
	F	M	F	M	F	M
**1924**	369.05 ± 10.95	314.88 ± 13.09	NA	NA	NA	1280.48 ± 53.60
**1928**	361.10 ± 10.29	309.37 ± 9.17	NA	NA	NA	1246.33 ± 45.84
**1932**	350.25 ± 18.90	292.73 ± 4.07	NA	NA	NA	1180.58 ± 23.89
**1936**	334.43 ± 9.61	291.31 ± 5.70	NA	NA	NA	1183.13 ± 16.56
**1948**	325.56 ± 4.96	289.64 ± 5.89	NA	NA	NA	1200.24 ± 28.74
**1952**	317.93 ± 5.32	277.19 ± 4.32	NA	NA	NA	1139.94 ± 25.00
**1956**	310.50 ± 7.90	274.06 ± 4.46	NA	NA	NA	1106.85 ± 23.21
**1960**	297.78 ± 4.02	263.50 ± 3.69	NA	NA	NA	1062.59 ± 21.36
**1964**	288.94 ± 3.80	257.06 ± 3.05	NA	NA	NA	1037.29 ± 11.68
**1968**	281.20 ± 7.84	255.99 ± 5.09	577.96 ± 13.54	NA	NA	1039.08 ± 28.3
**1972**	264.71 ± 4.02	244.03 ± 2.73	542.63 ± 9.67^j^	NA	NA	974.86 ± 14.69
**1976**	255.06 ± 3.55	237.05 ± 3.71	525.41 ± 5.76^j,k^	NA	NA	921.23 ± 16.19
**1980**	253.22 ± 3.69	234.88 ± 2.06	518.95 ± 6.69^j,k^	NA	NA	917.62 ± 9.50
**1984**	252.98 ± 3.34	233.48 ± 1.82	518.37 ± 8.31^j,k^	NA	NA	921.62 ± 11.52
**1988**	249.18 ± 3.75^a-e^	228.66 ± 1.55^a-e^	508.58 ± 7.74^j-l^	NA	NA	913.36 ± 12.15
**1992**	251.54 ± 3.06	227.83 ± 1.97^a-e^	514.79 ± 5.40^j,k^	NA	NA	908.90 ± 15.85^a,b,e^ ^e^
**1996**	250.07 ± 2.17	230.57 ± 2.03	514.89 ± 5.49^j,k^	NA	NA	910.29 ± 9.18^a,b,e^
**2000**	249.46 ± 2.55^a-e^	226.21 ± 2.76^a-f^	507.82 ± 5.74^j-l^	NA	NA	901.67 ± 10.15^a-f^
**2004**	248.05 ± 2.57^a-f^	225.92 ± 2.26^a-f^	509.06 ± 4.39^j-l^	NA	NA	898.05 ± 12.62^a-f^
**2008**	244.90 ± 2.66^a-h^	223.72 ± 1.98^a-j^	504.04 ± 5.38^j-n^	NA	NA	889.11 ± 9.63^a-h^
**2012**	244.14 ± 1.94^a-i^	225.53 ± 3.13^a-g^	502.84 ± 5.00^j-n^	NA	NA	887.70 ± 9.76^a-h^
**2016**	243.08 ± 3.34^a-i^	224.24 ± 2.36^a-h^	498.68 ± 6.85^j-n,p,q^	NA	NA	886.81 ± 8.81^a-h,j^
**2020**	242.68 ± 4.13^a-i^	224.30 ± 0.76^a-h^	499.9 ± 4.80 ^j-n,p,q^	465.19 ± 4.08	951.07 ± 10.48	890.66 ± 10.33^a-h^
**2024**	241.34 ± 2.49^a-j^	223.72 ± 2.03^a-i^	496.98 ± 4.93 ^j-n,p,q^	462.61 ± 3.64	951.84 ± 14.82	880.95 ± 6.59^a-j^

*Data are presented as mean*±SD. F: Female, M: Male, *NA: Not available. Each letter shown as a superscript denotes a significant difference with an Olympic Games. The letters respectively represent the following Olympics: a: 1924, b: 1928, c: 1932, d: 1936, e: 1948, f: 1952, g: 1956, h: 1960, i: 1964, j: 1968, k: 1972, l: 1976, m: 1980, n: 1984, o: 1988, p: 1992, q: 1996, r: 2000, s: 2004, t: 2008, u: 2012, v: 2016, w: 2020, x: 2024.*

The 800-m freestyle final time of female swimmers decreased significantly from the 1968 Olympics to the 2024 Olympic Games (577.96 ± 13.54 vs. 496.98 ± 4.93 sec), corresponding to a total reduction of 80.98 sec (14.0%) (Table 2). The largest percentage reduction in 800-m freestyle final time among women was recorded at the 1972 Olympic Games (6.1%). Men’s 800-m freestyle was first introduced at the 2020 Olympic Games, and no significant differences were observed in final times between the performances recorded at the 2000 and 2024 Games.

Men’s 1500-m freestyle final times decreased significantly from the 1924 to the 2024 Olympic Games (1280.48 ± 53.6 vs. 880.95 ± 6.59 sec), corresponding to a total reduction of 399.53 sec (31.2%; Table 2). The largest percentage reductions were observed in men in 1972 (6.2%) and 1976 (5.5%). In women, the 1500-m freestyle event was introduced for the first time at the 2020 Olympic Games, and no significant differences were found in final times between the 2000 and 2024 Olympic Games.

**Table 3:** Final performance swimming speeds of swimmers in the 50-, 100-, and 200-meter freestyle events at the Olympic Games (1924–2024) (m.sec^-1^).

	50-m		100-m		200-m	
	F	M	F	M	F	M
**1924**	NA	NA	1.333 ± 0.058	1.628 ± 0.040	NA	NA
**1928**	NA	NA	1.365 ± 0.032	1.655 ± 0.029	NA	NA
**1932**	NA	NA	1.454 ± 0.031	1.697 ± 0.014	NA	NA
**1936**	NA	NA	1.484 ± 0.024	1.706 ± 0.028	NA	NA
**1948**	NA	NA	1.473 ± 0.023	1.706 ± 0.031^a,b^	NA	NA
**1952**	NA	NA	1.484 ± 0.013	1.716 ± 0.017^a,b^	NA	NA
**1956**	NA	NA	1.548 ± 0.038	1.753 ± 0.031^a-f^	NA	NA
**1960**	NA	NA	1.574 ± 0.033	1.788 ± 0.022^a-f^	NA	NA
**1964**	NA	NA	1.627 ± 0.033	1.841 ± 0.029^a-h^	NA	NA
**1968**	NA	NA	1.646 ± 0.016	1.874 ± 0.022^a-h^	1.504 ± 0.024	1.689 ± 0.034
**1972**	NA	NA	1.682 ± 0.016	1.917 ± 0.022^a-j^	1.579 ± 0.035	1.739 ± 0.025
**1976**	NA	NA	1.763 ± 0.016	1.945 ± 0.028^a-j^	1.629 ± 0.028	1.796 ± 0.018
**1980**	NA	NA	1.762 ± 0.042	1.944 ± 0.023^a-j^	1.641 ± 0.027	1.787 ± 0.019
**1984**	NA	NA	1.765 ± 0.023	1.982 ± 0.014^a-m^	1.660 ± 0.017	1.810 ± 0.032
**1988**	1.941 ± 0.012	2.199 ± 0.033	1.793 ± 0.017	2.009 ± 0.027^a-m^	1.671 ± 0.022	1.846 ± 0.011
**1992**	1.972 ± 0.027	2.234 ± 0.027	1.803 ± 0.022^a,b^	2.017 ± 0.014^a-m^	1.668 ± 0.020	1.849 ± 0.021
**1996**	1.972 ± 0.029	2.226 ± 0.024	1.806 ± 0.019^a,b^	2.028 ± 0.018^a-n^	1.668 ± 0.018	1.845 ± 0.008
**2000**	1.999 ± 0.038^o^	2.254 ± 0.020^o^	1.827 ± 0.021^a-f^	2.043 ± 0.016^a-n^	1.682 ± 0.007^j^	1.862 ± 0.026^j^
**2004**	2.003 ± 0.015^o^	2.261 ± 0.016^o-q^	1.832 ± 0.016^a-f^	2.049 ± 0.020^a-o^	1.685 ± 0.006^j^	1.878 ± 0.024^j,k^
**2008**	2.053 ± 0.024^o-s^	2.318 ± 0.015^o-s^	1.859 ± 0.017^a-j^	2.093 ± 0.017^a-s^	1.719 ± 0.020^j-m^	1.891 ± 0.027^j-m^
**2012**	2.045 ± 0.018^o-s^	2.306 ± 0.021^o-s^	1.869 ± 0.010^a-j^	2.089 ± 0.013^a-s^	1.720 ± 0.020^j-m^	1.894 ± 0.026^j-m^
**2016**	2.064 ± .018^o-s^	2.307 ± 0.025^o-s^	1.885 ± 0.009^a-j^	2.085 ± 0.011^a-s^	1.737 ± 0.014^j-n^	1.896 ± 0.008^j-m^
**2020**	2.064 ± .017^o-s^	2.315 ± 0.025^o-s^	1.901 ± 0.014^a-k^	2.099 ± 0.018^a-s^	1.739 ± 0.015^j-n^	1.907 ± 0.010^j-n^
**2024**	2.067 ± 0.021^o-s^	2.324 ± 0.017^o-s^	1.902 ± 0.012^a-k^	2.104 ± 0.023^a-s^	1.739 ± 0.016^j-n^	1.901 ± 0.010^j-n^

*Data are presented as mean*±SD. F: Female, M: Male, *NA: Not available. Each letter shown as a superscript denotes a significant difference with an Olympic Games. The letters respectively represent the following Olympics: a: 1924, b: 1928, c: 1932, d: 1936, e: 1948, f: 1952, g: 1956, h: 1960, i: 1964, j: 1968, k: 1972, l: 1976, m: 1980, n: 1984, o: 1988, p: 1992, q: 1996, r: 2000, s: 2004, t: 2008, u: 2012, v: 2016, w: 2020, x: 2024.*

### Freestyle swimming speed

Freestyle swimming speeds have increased significantly over time (Tables 3 and 4). The percentage increases in mean swimming speed from the first to the most recent Olympic Games were as follows: 50 m, 6.5% vs. 5.7%; 100 m, 42.7% vs. 29.2%; 200-m, 15.6% vs. 12.6%; and 400-m, 52.7% vs. 40.6% in women and men, respectively. In addition, the percentage of mean swimming speed difference was 16.3% in women for the 800-m event and 45.2% in men for the 1500-m event. As race distance increased, swimming speed decreased in both women and men. The greatest increases in freestyle speed were observed in women in the 100- and 800-m events and in men in the 1500-m event.

**Table 4:** Final performance swimming speeds of swimmers in the 400-, 800-, and 1500-meter freestyle events at the Olympic Games (1924–2024) (m.sec^-1^).

	400-m		800-m		1500-m	
	F	M	F	M	F	M
**1924**	1.085 ± 0.031	1.272 ± 0.052	NA	NA	NA	1.173 ± 0.050
**1928**	1.108 ± 0.032	1.294 ± 0.037	NA	NA	NA	1.205 ± 0.044
**1932**	1.145 ± 0.062	1.367 ± 0.019^a,b^	NA	NA	NA	1.271 ± 0.026
**1936**	1.197 ± 0.035	1.374 ± 0.027^a,b^	NA	NA	NA	1.268 ± 0.018
**1948**	1.229 ± 0.019	1.382 ± 0.028^a,b^	NA	NA	NA	1.250 ± 0.030
**1952**	1.258 ± 0.021	1.443 ± 0.023^a-e^	NA	NA	NA	1.316 ± 0.028
**1956**	1.289 ± 0.033	1.460 ± 0.024^a-e^	NA	NA	NA	1.356 ± 0.028
**1960**	1.344 ± 0.018	1.518 ± 0.021^a-g^	NA	NA	NA	1.412 ± 0.028
**1964**	1.385 ± 0.018	1.556 ± 0.019^a-h^	NA	NA	NA	1.446 ± 0.016
**1968**	1.423 ± 0.039	1.563 ± 0.031^a-h^	1.385 ± 0.032	NA	NA	1.445 ± 0.039
**1972**	1.511 ± 0.023	1.639 ± 0.018^a-j^	1.475 ± 0.026^j^	NA	NA	1.539 ± 0.023
**1976**	1.569 ± 0.022	1.688 ± 0.026^a-k^	1.523 ± 0.017^j,k^	NA	NA	1.629 ± 0.029
**1980**	1.580 ± 0.023	1.703 ± 0.015^a-k^	1.542 ± 0.020^j,k^	NA	NA	1.635 ± 0.017
**1984**	1.581 ± 0.021	1.713 ± 0.013^a-k^	1.544 ± 0.025^j,k^	NA	NA	1.628 ± 0.020
**1988**	1.606 ± 0.024^a-e^	1.749 ± 0.012^a-m^	1.573 ± 0.024^j-l^	NA	NA	1.643 ± 0.022
**1992**	1.590 ± 0.019^a-e^	1.756 ± 0.015^a-n^	1.554 ± 0.016^j-k^	NA	NA	1.651 ± 0.029^a,b,e^
**1996**	1.600 ± 0.014^a-e^	1.735 ± 0.015^a-l^	1.554 ± 0.017^j-k^	NA	NA	1.648 ± 0.017^a,b,e^
**2000**	1.604 ± 0.016^a-e^	1.768 ± 0.022^a-n^	1.576 ± 0.018^j-l^	NA	NA	1.664 ± 0.019^a-f^
**2004**	1.613 ± 0.017^a-f^	1.771 ± 0.018^a-n^	1.572 ± 0.014^j-l^	NA	NA	1.671 ± 0.024^a-f^
**2008**	1.634 ± 0.017^a-h^	1.788 ± 0.016^a-o,q^	1.587 ± 0.017^j-n^	NA	NA	1.703 ± 0.046^a-h^
**2012**	1.638 ± 0.013^a-i^	1.774 ± 0.025^a-n,q^	1.591 ± 0.016^j-n,p,q^	NA	NA	1.690 ± 0.019^a-h^
**2016**	1.646 ± 0.023^a-i^	1.784 ± 0.019^a-n,q^	1.605 ± 0.022^j-n,p,q^	NA	NA	1.692 ± 0.017^a-h,j^
**2020**	1.649 ± 0.028^a-i^	1.783 ± 0.006^a-n,q^	1.600 ± 0.016^j-n,p,q^	1.720 ± 0.015	1.577 ± 0.017	1.692 ± 0.017^a-h^
**2024**	1.657 ± 0.017^a-j^	1.788 ± 0.016^a-n,q^	1.610 ± 0.016^j-n,p,q^	1.729 ± 0.014	1.576 ± 0.025	1.703 ± 0.013^a-h^

*Data are presented as mean*±SD. F: Female, M: Male, *NA: Not available. Each letter shown as a superscript denotes a significant difference with an Olympic Games. The letters respectively represent the following Olympics: a: 1924, b: 1928, c: 1932, d: 1936, e: 1948, f: 1952, g: 1956, h: 1960, i: 1964, j: 1968, k: 1972, l: 1976, m: 1980, n: 1984, o: 1988, p: 1992, q: 1996, r: 2000, s: 2004, t: 2008, u: 2012, v: 2016, w: 2020, x: 2024.*

### Freestyle reaction time

Reaction-time data were available from the 2000 Olympic Games onwards. Within this period, freestyle reaction times showed a significant decrease from the 2000 to the 2024 Olympic Games (Table 5). The fastest reaction times in both sexes were recorded in the 50-m freestyle event (0.67 ± 0.05 sec in women and 0.62 ± 0.03 sec in men).

**Table 5:** Reaction times of swimmers in freestyle final performances at the Olympic Games (1924–2024) (sec).

	50-m		100-m		200-m	
	F	M	F	M	F	M
**2000**	0.75 ± 0.04	0.79 ± 0.07	0.74 ± 0.06	0.79 ± 0.05	0.85 ± 0.05	0.80 ± 0.06
**2004**	0.75 ± 0.04	0.69 ± 0.07^a^	0.75 ± 0.04	0.74 ± 0.06	0.84 ± 0.05	0.79 ± 0.06
**2008**	0.76 ± 0.04	0.70 ± 0.05^a^	0.78 ± 0.07	0.71 ± 0.04^a^	0.78 ± 0.06	0.73 ± 0.06
**2012**	0.71 ± 0.03^a-c^	NA	0.73 ± 0.03	0.69 ± 0.03^a^	0.72 ± 0.04^a,b^	0.70 ± 0.04^a,b^
**2016**	0.68 ± .05^a-c^	0.66 ± 0.05^a^	0.70 ± 0.04	0.67 ± 0.04^a,b^	0.71 ± 0.04^a,b^	0.70 ± 0.08^a,b^
**2020**	0.67 ± .05^a-c^	0.63 ± 0.02^a^	0.67 ± 0.07^c^	0.63 ± 0.04^a-c^	0.69 ± 0.05 ^a-c^	0.66 ± 0.04^a,b^
**2024**	0.68 ± .04^a-c^	0.62 ± 0.03^a,c^	0.68 ± 0.04^c^	0.65 ± 0.03^a,b^	0.70 ± 0.04^a,b^	0.68 ± 0.04^a,b^
	**400-m**		**800-m**		**1500-m**	
	**F**	**M**	**F**	**M**	**F**	**M**
**2000**	0.85 ± 0.09	0.82 ± 0.04	0.85 ± 0.09	NA	NA	0.84 ± 0.08
**2004**	0.86 ± 0.06	0.83 ± 0.07	0.86 ± 0.06	NA	NA	0.83 ± 0.09
**2008**	0.81 ± 0.05	0.76 ± 0.04^a,b^	0.81 ± 0.05	NA	NA	0.81 ± 0.05
**2012**	0.73 ± 0.03^a-c^	0.71 ± 0.03^a,b^	0.73 ± 0.02	NA	NA	0.79 ± 0.05
**2016**	0.72 ± 0.04^a-c^	0.72 ± 0.04^a,b^	0.73 ± 0.04^a,b^	NA	NA	0.74 ± 0.05
**2020**	0.72 ± 0.05^a-c^	0.71 ± 0.03^a,b^	0.72 ± 0.04 ^a-c^	0.73 ± 0.03	0.72 ± 0.07	0.73 ± 0.05^a^
**2024**	0.73 ± 0.03^a-c^	0.67 ± 0.03^a-c^	0.73 ± 0.04^a,b^	0.71 ± 0.05	0.73 ± 0.04	0.74 ± 0.04

*Data are presented as mean*±SD. F: Female, M: Male, *NA: Not available. Reaction time data prior to the 2000 Olympic Games are not available. Each letter shown as a superscript denotes a significant difference with an Olympic Games. The letters respectively represent the following Olympics: a:2000, b: 2004, c: 2008, d: 2012, e: 2016, f: 2020, g: 2024.*

### Backstroke final time and swimming speed

From 1924 to the 2024 Olympic Games, 100-m backstroke final times showed a significant decline (88.16 ± 3.22 vs. 58.59 ± 0.89 sec in women, and 79.16 ± 6.36 vs. 52.51 ± 0.28 sec in men), corresponding to total reductions of 29.57 sec (33.5%) in women and 26.65 sec (33.7%) in men (Table 6). The largest percentage reductions in 100-m backstroke final times were observed in women and men in the 1928 Olympics (5.1% vs. 8.8%) and again in the 1956 Olympics (3.2% vs. 3.6%). A notable increase in race time among women (4.8%) occurred in the 1984 Olympic Games.

**Table 6:** Backstroke final performance times (sec) and swimming speeds (m.sec^-1^) of swimmers at the Olympic Games (1924–2024).

	Final time				Swimming speed			
	100-m		200-m		100-m		200-m	
	F	M	F	M	F	M	F	M
**1924**	88.16 ± 3.22	79.16 ± 6.36	NA	NA	1.136 ± 0.04	1.269 ± 0.096	NA	NA
**1928**	83.69 ± 1.39	72.17 ± 2.67	NA	NA	1.195 ± 0.020	1.387 ± 0.052	NA	NA
**1932**	82.07 ± 1.5	70.57 ± 1.44	NA	NA	1.219 ± 0.023	1.418 ± 0.029	NA	NA
**1936**	79.94 ± .92	68.89 ± 1.69	NA	NA	1.251 ± 0.014	1.452 ± 0.036	NA	NA
**1948**	77.66 ± 1.75	68.33 ± 1.27	NA	NA	1.288 ± 0.030	1.464 ± 0.028	NA	NA
**1952**	76.14 ± 1.58	67.19 ± 1.05	NA	NA	1.314 ± 0.27	1.489 ± 0.023	NA	NA
**1956**	73.71 ± .72	64.76 ± 1.49	NA	NA	1.357 ± 0.013	1.545 ± 0.036	NA	NA
**1960**	71.33 ± .95	63.15 ± .97	NA	NA	1.402 ± 0.019	1.584 ± 0.024	NA	NA
**1964**	69.01 ± 1.19	NA	NA	NA	1.449 ± 0.025	NA	NA	NA
**1968**	68.43 ± 1.44	60.95 ± 1.12	150.86 ± 3.88	133.04 ± 2.61	1.462 ± 0.031	1.641 ± 0.031	1.326 ± 0.034	1.504 ± 0.030
**1972**	66.65 ± .53	58.84 ± 1.30	142.97 ± 2.16^j^	126.96 ± 3.13	1.500 ± 0.012	1.700 ± 0.038	1.399 ± 0.021^j^	1.576 ± 0.039
**1976**	64.26 ± 1.28	57.23 ± .93	136.35 ± 1.59^j,k^	123.71 ± 3.25	1.557 ± 0.031	1.748 ± 0.029	1.467 ± 0.017^j,k^	1.618 ± 0.043
**1980**	63.33 ± 1.38	57.63 ± .59	135.20 ± 1.93^j,k^	123.67 ± 1.29	1.580 ± 0.035	1.735 ± 0.018	1.480 ± 0.021^j,k^	1.617 ± 0.017
**1984**	66.34 ± .69	56.95 ± .72	134.85 ± 1.89^j,k^	122.95 ± 1.49	1.579 ± 0.017	1.756 ± 0.022	1.483 ± 0.021^j,k^	1.627 ± 0.020
**1988**	62.65 ± 1.23	55.69 ± .53	132.92 ± 2.39^j-l^	121.02 ± 1.15	1.597 ± 0.031	1.796 ± 0.017	1.505 ± 0.027^j-l^	1.653 ± 0.016
**1992**	61.81 ± .78^a-d^	55.12 ± .83^a-c^	131.23 ± 2.21^j-m^	119.59 ± .74	1.618 ± 0.020^a-d^	1.814 ± 0.027^a-c^	1.524 ± 0.026^j-m^	1.672 ± 0.010
**1996**	62.20 ± .58^a,b^	55.18 ± .51^a-c^	132.42 ± 2.42^j-l^	120.88 ± 3.07	1.608 ± 0.015^a,b^	1.812 ± 0.017^a-c^	1.511 ± 0.026^j-l^	1.655 ± 0.040
**2000**	61.14 ± .58^a-e^	54.85 ± .66^a-e^	131.71 ± 1.99^j-m^	118.43 ± 1.02^j^	1.636 ± 0.016^a-e^	1.823 ± 0.022^a-e^	1.519 ± 0.023^j-m^	1.689 ± 0.015
**2004**	61.07 ± .48^a-e^	54.52 ± .35^a-f^	130.69 ± 1.29^j-n^	117.98 ± 1.52^j,k^	1.637 ± 0.013^a-e^	1.834 ± 0.012^a-f^	1.530 ± 0.015^j-n^	1.695 ± 0.022^j,k^
**2008**	59.53 ± .43^a-h^	53.28 ± .41^a-h^	127.77 ± 1.54^j-r^	115.54 ± 1.09^j-n^	1.680 ± 0.012^a-h^	1.877 ± 0.014^a-h^	1.565 ± 0.019^j-r^	1.731 ± 0.016^j-n^
**2012**	59.13 ± .64^a-i^	53.16 ± .50^a-h^	127.21 ± 1.75^j-s^	115.62 ± 1.81^j-n^	1.691 ± 0.018^a-i^	1.881 ± 0.018^a-h^	1.572 ± 0.022^j-s^	1.730 ± 0.027^j-n^
**2016**	58.86 ± .26^a-j^	52.68 ± .54^a-h,j^	127.85 ± 1.29^j-r^	115.09 ± 1.10^j-n^	1.699 ± 0.007^a-j^	1.898 ± 0.019^a-h,j^	1.565 ± 0.016^j-r^	1.738 ± 0.017^j-n^
**2020**	58.43 ± .69^a-j^	52.44 ± .38^a-h,j^	126.76 ± 1.42^j-s^	115.82 ± 1.84^j-n^	1.712 ± 0.020^a-j^	1.907 ± 0.014^a-h,j^	1.578 ± 0.018^j-s^	1.727 ± 0.027^j-n^
**2024**	58.59 ± .89^a-j^	52.51 ± .28^a-h,j^	126.63 ± 2.21^j-s^	115.22 ± .54^j-n^	1.707 ± 0.026^a-j^	1.905 ± 0.010^a-h,j^	1.580 ± 0.027^j-s^	1.736 ± 0.008^j-n^

*Data are presented as mean*±SD. F: Female, M: Male, *NA: Not available. Each letter shown as a superscript denotes a significant difference with an Olympic Games. The letters respectively represent the following Olympics: a: 1924, b: 1928, c: 1932, d: 1936, e: 1948, f: 1952, g: 1956, h: 1960, i: 1964, j: 1968, k: 1972, l: 1976, m: 1980, n: 1984, o: 1988, p: 1992, q: 1996, r: 2000, s: 2004, t: 2008, u: 2012, v: 2016, w: 2020, x: 2024.*

Swimmers’ 200-m backstroke final times decreased significantly from the 1968 Olympic Games to the 2024 Olympic Games (150.86 ± 3.88 vs. 126.63 ± 2.21 sec in women, and 133.04 ± 2.61 vs. 115.22 ± 0.54 sec in men), corresponding to total reductions of 24.23 sec (16.1%) in women and 17.82 sec (13.4%) in men (Table 6). The largest percentage reductions in 200-m backstroke final times were observed in both women and men in the 1972 Olympic Games (5.2% vs. 4.6%) and again in the 2008 Games (2.2% vs. 2.1%).

The backstroke swimming speeds of athletes increased significantly over time (Table 6). The magnitude of improvement between early and recent Olympic Games was 50.3% in women and 50.1% in men for the 100-m event, and 19.2% in women and 15.4% in men for the 200-m event. In both sexes, swimming speed decreased as race distance increased.

### Breaststroke final time and swimming speed

Swimmers’ 100-m breaststroke final times decreased significantly from the 1968 to the 2024 Olympic Games (76.53 ± 0.60 vs. 65.81 ± 0.46 sec in women, and 68.40 ± 0.62 vs. 59.29 ± 0.32 sec in men), with total reductions of 10.72 sec (14%) in women and 9.11 sec (13.3%) in men (Table 7). The largest percentage reductions in 100-m breaststroke final times were observed in women during the 1976 (3.5%) and 1980 (2.7%) Olympic Games, and in men during the 1972 (3.5%), 1976 (2.8%), and 2008 (2.5%) Games.

**Table 7:** Breaststroke final performance times (sec) and swimming speeds (m.sec^-1^) of swimmers at the Olympic Games (1924–2024).

	Final time				Swimming speed			
	100-m		200-m		100-m		200-m	
	F	M	F	M	F	M	F	M
**1924**	NA	NA	217.86 ± 4.82	180.92 ± 3.36	NA	NA	0.918 ± 0.020	1.106 ± 0.021
**1928**	NA	NA	197.77 ± 3.59	174.4 ± 3.69^a^	NA	NA	1.012 ± 0.02	1.147 ± 0.025^a^
**1932**	NA	NA	191.07 ± 4.77	168.18 ± 2.60^a,b^	NA	NA	1.047 ± 0.026	1.189 ± 0.018^a,b^
**1936**	NA	NA	188.16 ± 3.27	165.76 ± 3.30^a-c^	NA	NA	1.063 ± 0.019	1.207 ± 0.024^a-c^
**1948**	NA	NA	182.49 ± 4.24	164.59 ± 3.21^a-c^	NA	NA	1.092 ± 0.026	1.216 ± 0.024^a-c^
**1952**	NA	NA	177.49 ± 4.0	157.5 ± 2.26^a-e^	NA	NA	1.127 ± 0.026	1.270 ± 0.018^a-e^
**1956**	NA	NA	175.93 ± 1.79	158.24 ± 2.44^a-e^	NA	NA	1.137 ± 0.012	1.264 ± 0.019^a-e^
**1960**	NA	NA	173.59 ± 2.47	159.76 ± 1.41^a-e^	NA	NA	1.152 ± 0.017	1.252 ± 0.011^a-e^
**1964**	NA	NA	169.68 ± 2.36	151.80 ± 3.55^a-f^	NA	NA	1.179 ± 0.016	1.318 ± 0.030^a-f^
**1968**	76.53 ± .6	68.40 ± .62	168.14 ± 2.53	151.58 ± 2.32^a-f^	1.307 ± 0.010	1.462 ± 0.013	1.190 ± 0.018	1.320 ± 0.020^a-f^
**1972**	76.09 ± 1.31	66.04 ± .37^j^	163.17 ± 1.18	145.12 ± 2.08^a-j^	1.315 ± 0.023	1.514 ± 0.008^j^	1.226 ± 0.009	1.378 ± 0.020^a-j^
**1976**	73.45 ± 1.04^j,k^	64.19 ± .65^j,k^	156.85 ± 1.79	139.79 ± 2.58^a-k^	1.362 ± 0.020^j,k^	1.558 ± 0.016^j,k^	1.275 ± 0.015	1.431 ± 0.027^a-k^
**1980**	71.48 ± .84^j-l^	64.39 ± .87^j,k^	152.72 ± 2.17	139.26 ± 2.34^a-k^	1.399 ± 0.016^j-l^	1.553 ± 0.021^j,k^	1.310 ± 0.019	1.437 ± 0.024^a-k^
**1984**	70.99 ± .59^j-l^	63.09 ± .87^j-m^	152.98 ± 1.87	137.58 ± 2.4^a-l^	1.409 ± 0.012^j-l^	1.585 ± 0.022^j-m^	1.308 ± 0.016	1.454 ± 0.026^a-l^
**1988**	69.30 ± .93^j-n^	62.51 ± .47^j-m^	148.55 ± 1.08	135.67 ± 1.35^a-n^	1.443 ± 0.019^j-n^	1.600 ± 0.012^j-m^	1.346 ± 0.010	1.474 ± 0.015^a-n^
**1992**	69.57 ± 1.06^j-n^	61.92 ± .29^j-n^	148.98 ± 2.05^a,b^	132.84 ± 1.76^a-o^	1.438 ± 0.022^j-n^	1.615 ± 0.08^j-n^	1.343 ± 0.019^a,b^	1.506 ± 0.020^a-o^
**1996**	69.02 ± .72^j-n^	61.46 ± .53^j-o^	147.73 ± 1.91^a-d^	134.31 ± 1.27^a-n^	1.449 ± 0.015^j-n^	1.627 ± 0.014^j-o^	1.354 ± 0.017^a-d^	1.489 ± 0.014^a-n^
**2000**	68.17 ± .92^j-n,p^	61.25 ± .49^j-o^	145.64 ± 1.05^a-f^	132.85 ± .92^a-o^	1.467 ± 0.020^j-n,p^	1.633 ± 0.013^j-o^	1.373 ± 0.009^a-f^	1.506 ± 0.011^a-o^
**2004**	67.39 ± .48^j-q^	61.15 ± .75^j-o^	145.49 ± 1.26^a-f^	131.14 ± .89^a-q^	1.484 ± 0.011^j-q^	1.636 ± 0.020^j-o^	1.375 ± 0.012^a-f^	1.525 ± 0.010^a-q^
**2008**	67.30 ± .9^j-q^	59.64 ± .47^j-s^	143.30 ± 1.63^a-h^	129.44 ± 1.17^a-s^	1.486 ± 0.022^j-q^	1.677 ± 0.013^j-s^	1.396 ± 0.016^a-h^	1.545 ± 0.014^a-s^
**2012**	66.61 ± .74^j-r^	59.61 ± .71^j-s^	142.38 ± 2.05^a-i^	128.54 ± .85^a-s^	1.502 ± 0.017^j-r^	1.678 ± 0.020^j-s^	1.405 ± 0.020^a-i^	1.556 ± 0.010^a-s^
**2016**	66.47 ± 1.06^j-r^	58.99 ± .84^j-s^	142.37 ± 1.01^a-i^	127.81 ± .28^a-s^	1.505 ± 0.024^j-r^	1.696 ± 0.025^j-s^	1.405 ± 0.010^a-i^	1.565 ± 0.003^a-s^
**2020**	65.85 ± .62^j-t^	58.61 ± .66^j-u^	141.69 ± 1.91^a-j^	127.65 ± .84^a-t^	1.519 ± 0.014^j-t^	1.707 ± 0.019^j-u^	1.412 ± 0.019^a-j^	1.567 ± 0.010^a-t^
**2024**	65.81 ± .46^j-t^	59.29 ± .32^j-s^	142.61 ± 2.43^a-h^	128.45 ± 1.70^a-s^	1.519 ± 0.011^j-t^	1.687 ± 0.009^j-s^	1.403 ± 0.024^a-k^	1.557 ± 0.021^a-s^

*Data are presented as mean*±SD. F: Female, M: Male, *NA: Not available. Each letter shown as a superscript denotes a significant difference with an Olympic Games. The letters respectively represent the following Olympics: a: 1924, b: 1928, c: 1932, d: 1936, e: 1948, f: 1952, g: 1956, h: 1960, i: 1964, j: 1968, k: 1972, l: 1976, m: 1980, n: 1984, o: 1988, p: 1992, q: 1996, r: 2000, s: 2004, t: 2008, u: 2012, v: 2016, w: 2020, x: 2024.*

The 200-m breaststroke final time showed a significant decline from the 1924 to the 2024 Olympic Games (217.86 ± 4.82 vs. 142.61 ± 2.43 sec in women, and 180.92 ± 3.36 vs. 128.45 ± 1.70 sec in men), corresponding to total reductions of 75.25 sec (34.5%) in women and 52.47 sec (29.0%) in men (Table 7). The largest percentage reductions in 200-m breaststroke final times were recorded in women during the 1928 (9.2%) and 1976 (3.9%) Olympics, and in men during the 1964 (5.0%), 1952 (4.3%), and 1972 (4.3%) Olympic Games.

Breaststroke swimming speed has increased significantly from past to present (Table 7). The magnitude of improvement between the earliest and most recent Olympics was 16.2% in women and 15.4% in men for the 100-m event, and 52.8% in women and 40.8% in men for the 200-m event. As race distance increases, a decline in swimming speed is observed in both women and men.

### Butterfly final time and swimming speed

Swimmers’ 100-m butterfly final times showed a significant decrease from the 1956 Olympics to the 2024 Games in women, and from the 1968 Olympics to the 2024 Games in men (75.14 ± 2.72 vs. 56.47 ± 0.66 sec in women; 57.96 ± 1.35 vs. 50.59 ± 0.46 sec in men). The total reductions corresponded to 18.67 sec (24.8%) in women and 7.37 sec (12.7%) in men (Table 8). The largest percentage reductions in 100-m butterfly final times were observed in women at the 1964 (6.7%) and 1972 Olympics (4%), and in men at the 1972 (2.9%) and 1984 Olympics (2.4%).

**Table 8:** Butterfly final performance times (sec) and swimming speeds (m.sec^-1^) of swimmers at the Olympic Games (1924–2024).

	Final time				Swimming speed			
	100-m		200-m		100-m		200-m	
	F	M	F	M	F	M	F	M
**1924**	NA	NA	NA	NA	NA	NA	NA	NA
**1928**	NA	NA	NA	NA	NA	NA	NA	NA
**1932**	NA	NA	NA	NA	NA	NA	NA	NA
**1936**	NA	NA	NA	NA	NA	NA	NA	NA
**1948**	NA	NA	NA	NA	NA	NA	NA	NA
**1952**	NA	NA	NA	NA	NA	NA	NA	NA
**1956**	75.14 ± 2.72	NA	NA	146.10 ± 3.71	1.332 ± 0.049	NA	NA	1.370 ± 0.035
**1960**	72.23 ± 1.64	NA	NA	136.73 ± 2.39	1.385 ± 0.032	NA	NA	1.463 ± 0.026
**1964**	67.40 ± 1.84	NA	NA	130.71 ± 2.74	1.485 ± 0.041	NA	NA	1.531 ± 0.032
**1968**	66.56 ± .77	57.96 ± 1.35	148.59 ± 3.90	130.68 ± 1.64	1.503 ± 0.017	1.726 ± 0.040	1.347 ± 0.035	1.531 ± 0.019
**1972**	63.89 ± .31	56.27 ± 1.19	138.81 ± 2.74	123.84 ± 1.75	1.565 ± 0.007	1.778 ± 0.038	1.441 ± 0.028	1.615 ± 0.023
**1976**	61.46 ± .76	55.30 ± .85	133.25 ± 1.03	120.91 ± 1.76	1.627 ± 0.020	1.809 ± 0.028	1.501 ± 0.012	1.654 ± 0.024
**1980**	61.97 ± .94	55.29 ± .85	133.07 ± 2.15	121.89 ± 1.05	1.614 ± 0.025	1.808 ± 0.010	1.503 ± 0.024	1.641 ± 0.014
**1984**	61.22 ± .99	53.97 ± 1.04	132.38 ± 3.11	118.66 ± 1.49	1.634 ± 0.027	1.854 ± 0.036	1.512 ± 0.036	1.686 ± 0.021
**1988**	60.09 ± .80	53.61 ± .58	131.27 ± 1.20	118.88 ± 1.12	1.664 ± 0.022	1.866 ± 0.020	1.524 ± 0.014	1.682 ± 0.016
**1992**	59.61 ± .93^g^	53.82 ± .41	130.49 ± 1.76	118.57 ± 1.06	1.678 ± 0.026^g^	1.858 ± 0.014	1.533 ± 0.021	1.687 ± 0.015
**1996**	59.89 ± .64	53.08 ± .44	130.47 ± 1.38	117.97 ± .80^g^	1.670 ± 0.018	1.884 ± 0.016	1.533 ± 0.016	1.695 ± 0.012^g^
**2000**	58.48 ± .91^g-j^	52.53 ± .42^j^	128.19 ± 1.61^j,k^	116.69 ± 1.05^g-j^	1.710 ± 0.027^g-j^	1.904 ± 0.015^j^	1.560 ± 0.020^j,k^	1.714 ± 0.015^g-j^
**2004**	58.54 ± .73^g-j^	51.98 ± .57^j-m^	128.58 ± 1.72^j,k^	115.89 ± 1.17^g-j^	1.708 ± 0.021^g-j^	1.924 ± 0.021^j-m^	1.556 ± 0.021^j,k^	1.726 ± 0.018^g-j^
**2008**	57.70 ± .61^g-k^	51.23 ± .47^j-m^	126.58 ± 1.43^j-m^	113.86 ± 1.13^g-m^	1.733 ± 0.018^g,k^	1.952 ± 0.018^j-m^	1.580 ± 0.018^j-m^	1.757 ± 0.018^g-m^
**2012**	57.10 ± .54^g-k^	51.68 ± .28^j-m^	126.16 ± 1.23^j-n^	114.24 ± 1.01^g-m^	1.751 ± 0.016^g-k^	1.935 ± 0.011^j-m^	1.585 ± 0.016^j-n^	1.751 ± 0.016^g-m^
**2016**	56.63 ± .56^g-k,m^	51.29 ± .46^j-m^	126.40 ± 1.32^j-m^	114.77 ± 1.40^g-k,m^	1.766 ± 0.018^g-k,m^	1.950 ± 0.018^j-m^	1.582 ± 0.017^j-m^	1.743 ± 0.021^g-k,m^
**2020**	56.14 ± .58^g-n^	50.65 ± .71^j-p^	126.78 ± 1.79^j-m^	114.43 ± 1.44^g-k,m^	1.781 ± 0.018^g-n^	1.975 ± 0.028^j-p^	1.578 ± 0.022^j-m^	1.748 ± 0.022^g-k,m^
**2024**	56.47 ± .66^g-k,m^	50.59 ± .46^j-p^	126.36 ± 2.11^j-m^	113.42 ± 1.35^g-m^	1.771 ± 0.021^g-k,m^	1.977 ± 0.018^j-p^	1.583 ± 0.027^j-m^	1.764 ± 0.021 ^g-s^

*Data are presented as mean*±SD. F: Female, M: Male, *NA: Not available. Each letter shown as a superscript denotes a significant difference with an Olympic Games. The letters respectively represent the following Olympics: a: 1924, b: 1928, c: 1932, d: 1936, e: 1948, f: 1952, g: 1956, h: 1960, i: 1964, j: 1968, k: 1972, l: 1976, m: 1980, n: 1984, o: 1988, p: 1992, q: 1996, r: 2000, s: 2004, t: 2008, u: 2012, v: 2016, w: 2020, x: 2024.*

In the 200-m butterfly event, swimmers’ final times showed significant reductions from the 1968 Olympics in women and the 1956 Olympics in men through to the 2024 Olympic Games (148.59 ± 3.9 vs. 126.36 ± 2.11 sec in women, and 146.1 ± 3.71 vs. 113.42 ± 1.35 sec in men), corresponding to total reductions of 22.23 sec (15%) in women and 32.68 sec (22.4%) in men (Table 8). The largest percentage reductions in 200-m butterfly final times were observed in women in 1972 (6.6%) and 1976 (4.0%), and in men in 1960 (6.4%) and 1972 (5.2%).

Butterfly swimming speed showed significant increases from past to present (Table 8). In women and men, the magnitude of improvement between early and recent Olympics was 33.0% vs. 14.5% for 100 m and 17.5% vs. 28.8% for 200-m, respectively. In both sexes, swimming speed decreased as race distance increased.

### Backstroke & breaststroke & butterfly reaction time

Reaction times in backstroke, breaststroke, and butterfly showed significant decreases over the period for which data were available (Table 9). The fastest reaction times in both sexes were recorded in the 100-m event.

**Table 9:** Reaction times (sec) in backstroke, breaststroke, and butterfly final performances of swimmers at the Olympic Games (1924–2024).

	Backstroke				Breaststroke	
	100-m		200-m		100-m	
	F	M	F	M	F	M
**2000**	NA	NA	NA	NA	0.80 ± 0.05	0.76 ± 0.04
**2004**	0.67 ± 0.04	0.64 ± 0.07	0.69 ± 0.07	0.68 ± 0.06	0.75 ± 0.04	0.75 ± 0.04
**2008**	0.66 ± 0.08	0.62 ± 0.08	0.66 ± 0.04	0.67 ± 0.08	0.76 ± 0.05	0.76 ± 0.09
**2012**	0.63 ± 0.05	0.63 ± 0.04	0.67 ± 0.04	0.62 ± 0.05	0.69 ± 0.05^a^	0.68 ± 0.05
**2016**	0.66 ± 0.09	0.62 ± 0.07	0.67 ± 0.06	0.61 ± 0.04	0.68 ± 0.05^a,c^	0.66 ± 0.04^a^
**2020**	0.59 ± 0.05	0.57 ± 0.04	0.60 ± 0.03^b^	0.59 ± 0.04^b^	0.67 ± 0.03^a-c^	0.63 ± 0.02^a-c^
**2024**	0.59 ± 0.04	0.58 ± 0.06	0.59 ± 0.06^b^	0.60 ± 0.06	0.68 ± 0.02^a-c^	0.66 ± 0.04^a^
	**Breaststroke**		**Butterfly**			
	**200-m**		**100-m**		**200-m**	
	**F**	**M**	**F**	**M**	**F**	**M**
**2000**	0.79 ± 0.07	0.79 ± 0.04	0.79 ± 0.03	0.76 ± 0.03	0.81 ± 0.07	0.79 ± 0.03
**2004**	0.79 ± 0.04	0.79 ± 0.06	0.76 ± 0.04	0.75 ± 0.06	0.82 ± 0.07	0.78 ± 0.07
**2008**	0.79 ± 0.05	0.79 ± 0.06	0.76 ± 0.06	0.73 ± 0.03	0.76 ± 0.08	0.74 ± 0.03
**2012**	0.72 ± 0.05^a-c^	0.71 ± 0.05^a-c^	0.71 ± 0.04^a^	0.70 ± 0.05	0.74 ± 0.04	0.67 ± 0.04^a,b^
**2016**	0.75 ± 0.02	0.69 ± 0.03^a-c^	0.71 ± 0.03^a^	0.68 ± 0.05^a,b^	0.71 ± 0.04^a,b^	0.68 ± 0.05^a,b^
**2020**	0.69 ± 0.03^a-c^	0.65 ± 0.04^a-c^	0.67 ± 0.04^a-c^	0.63 ± 0.03^a-d^	0.68 ± 0.05^a,b^	0.65 ± 0.04^a-c^
**2024**	0.69 ± 0.05^a-c^	0.67 ± 0.02^a-c^	0.68 ± 0.05^a-c^	0.63 ± 0.02^a-d^	0.72 ± 0.04^b^	0.65 ± 0.03^a-c^

*Data are presented as mean*±SD. F: Female, M: Male, *NA: Not available. Each letter shown as a superscript denotes a significant difference with an Olympic Games. The letters respectively represent the following Olympics: a: 2000, b: 2004, c: 2008, d: 2012, e: 2016, f: 2020, g: 2024.*

## Discussion

Over the last century, swimming performance at the Olympic Games has undergone remarkable improvements, reflected by substantial reductions in final times across all strokes and distances. The findings of the present study indicate that the largest percentage improvements in performance were observed during specific Olympic cycles, including 1956, 1972, and 2008. These periods coincided with major technological, regulatory, and methodological developments in competitive swimming. For instance, the introduction of refined training systems, the evolution of hydrodynamic swimsuit designs, improvements in pool construction (lane lines, depth, water circulation), and stricter starting regulations likely contributed to substantial reductions in race times observed across freestyle, backstroke, breaststroke, and butterfly events. Although major technological, regulatory, and methodological developments occurred throughout the study period, the primary aim of the present investigation was to provide a comprehensive overview of long-term Olympic swimming performance across the entire 1924–2024 period rather than to formally compare predefined historical eras. Future studies may benefit from segmenting Olympic swimming history into distinct technological or regulatory periods to further examine the relative contribution of specific developments to performance progression. It should also be noted that the absence of statistically significant differences between some Olympic Games separated by several decades does not necessarily indicate an absence of long-term performance improvement. The significance of pairwise comparisons depends not only on the magnitude of differences between Olympic editions but also on within-edition variability and the multiple-comparison procedure applied. In addition, adjustment for multiple comparisons may result in some apparent differences between Olympic Games not reaching statistical significance. Therefore, chronological distance between Olympic Games should not be expected to correspond directly to statistical significance.

### Freestyle

In freestyle events, the Olympic cycles associated with the largest percentage improvements in performance (e.g., mid-1950s, early 1970s and the late 2000s) coincided with major developments in pool design and swimsuit technology (Fig 1). From the 1960s onward, Olympic pools became progressively deeper, wider and equipped with wave-lane ropes and energy-absorbing lane lines [20], all of which reduce turbulence and drag and are explicitly cited as reasons why modern pools are “faster” than earlier facilities. In parallel, the introduction and widespread adoption of low-profile competition goggles in the early 1970s improved underwater visibility, reduced ocular irritation, and allowed swimmers to maintain a more stable head position during extended training and racing sessions [21–23]. One of the largest percentage reductions in freestyle final times observed in our dataset occurred at the 2008 Olympic Games in both women (1.4%) and men (2.1%), coinciding with the widespread adoption of polyurethane-based full-body and panelled swimsuits. Previous studies have reported that these suits were associated with performance improvements of approximately 1.5–3.5% and distinct increases in world-record progression, particularly in low-resistance strokes such as freestyle [24,25].

**Fig 1:**
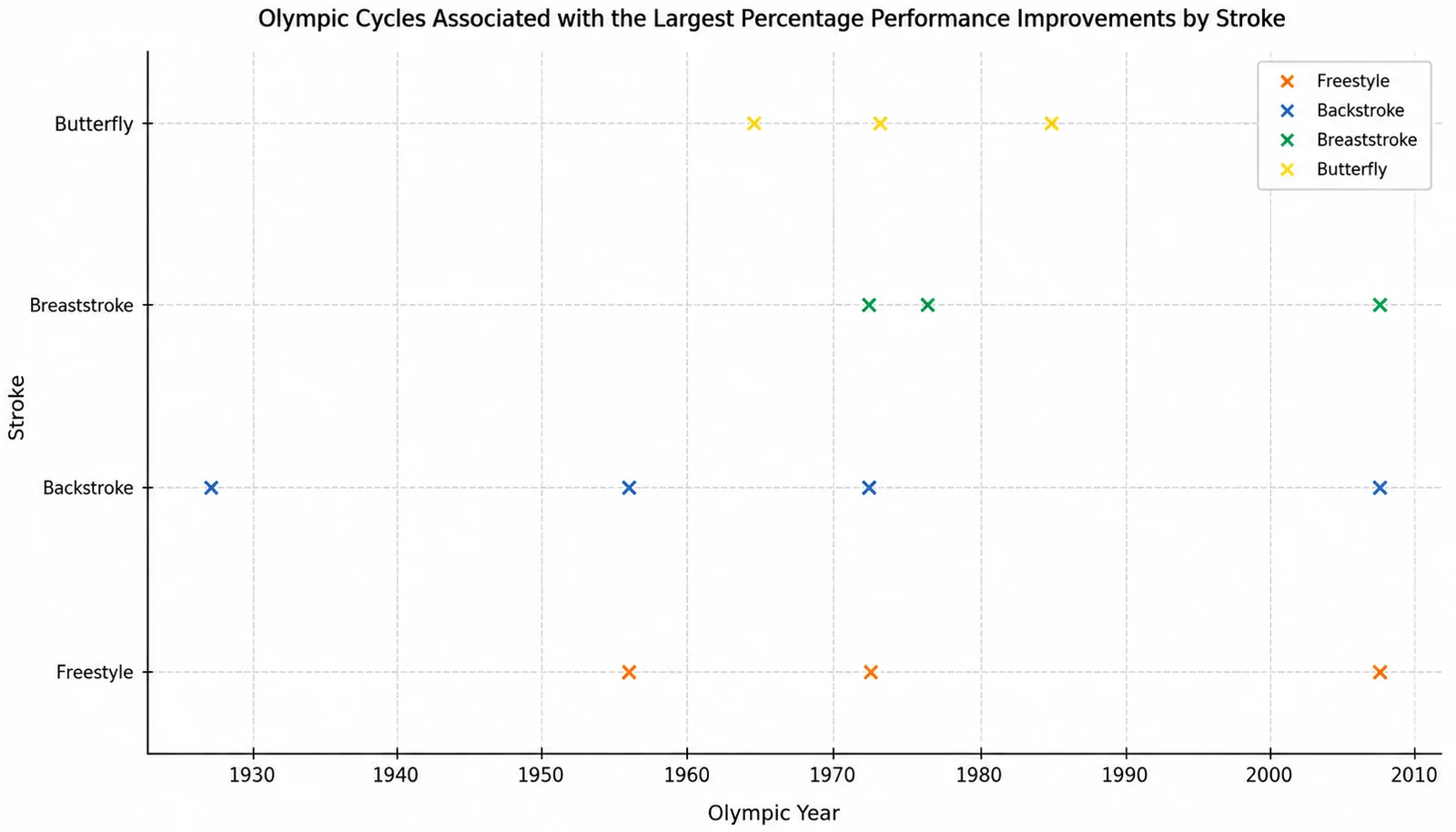
Olympic Games associated with the largest percentage improvements in performance across freestyle, backstroke, breaststroke, and butterfly events.

### Backstroke

For backstroke, the periods associated with the largest percentage improvements in performance were consistent with general advances in pool architecture, facility design, equipment technology, and stroke-specific rule modifications. During the 1980s, swimmers such as David Berkoff demonstrated that prolonged underwater dolphin kicking after the start and turns could substantially increase backstroke speed, prompting Fédération Internationale de Natation (FINA) to introduce and later extend the 15-m underwater limit in 1991 [26]. This regulatory change effectively codified the underwater phase as a key performance determinant and contributed to a more efficient exploitation of streamlined body positions and dolphin kicking in backstroke races [26,27]. At the same time, improvements in lane-rope technology and gutter systems reduced wave reflection and lane-to-lane interference, which may have further supported performance improvements in technically proficient swimmers [28,29]. One of the largest percentage improvements in backstroke performance was observed during the late 2000s, coinciding with the widespread use of high-tech swimsuits. Comparative analyses from this period reported significant reductions in race times in both freestyle and backstroke events during the polyurethane-suit era [25,30].

### Breaststroke

Breaststroke shows a somewhat different pattern: although our data still highlight specific Olympic cycles with large time reductions, the observed performance improvements may be more closely associated with changes in drag-related technology and stroke-specific technique than with underwater-rule modifications. Performance analyses have classified breaststroke and butterfly as “high-drag strokes,” in which improvements in suit materials and fit yield proportionally larger gains compared with freestyle and backstroke [25,30]. Studies evaluating high-tech swimsuits have indeed reported substantial performance benefits in these strokes, largely attributed to reduced form drag and improved body position at the surface [24,30]. In addition, the progressive refinement of stroke rules (e.g., clarifying arm recovery and kick patterns) and coaching emphasis on minimizing intra-cyclic speed fluctuations coincided with advances in pool design and race-analysis technology [31,32]. These developments occurred during Olympic Games in which some of the largest percentage reductions in breaststroke final times were observed in our dataset.

### Butterfly

Butterfly, as the youngest Olympic stroke, exhibited some of its largest percentage performance improvements during the decades following its formal separation from breaststroke and the stabilization of its technical rules. Early Olympic butterfly swimmers were still experimenting with arm recovery shape and kick timing [33]; over time, biomechanical analyses and underwater filming enabled a more standardized and efficient technique, especially in coupling the undulatory body motion with the dolphin kick [34,35]. The late 1960s and 1970s, during which our data indicate some of the largest percentage improvements in butterfly performance also coincide with the introduction of competition goggles [21–23] and more sophisticated pool environments [28,29], which have been associated with improved head-and-body alignment and reduced wave drag during the stroke cycle. Later, the extension of the 15-m underwater rule to butterfly in 1998 formalized the importance of the underwater dolphin-kick phase [26], whereas the polyurethane-suit era around 2008–2009 coincided with additional improvements in butterfly performance, consistent with observations reported in the literature on swimming technology and performance [24].

### Reaction Time

Reaction-time data were available only for the Olympic Games held between 2000 and 2024. Within this period, reaction times generally decreased across most swimming events, indicating improvements in start performance among elite swimmers. Although reaction time represents only a small component of total race duration, its contribution may be particularly important in sprint events where performance differences are often measured in hundredths of a second. The observed reductions in reaction time may be associated with refinements in start techniques, developments in competition technology, and increased emphasis on start-specific training practices within modern swimming programs [26,31]. Consequently, the observed improvements in reaction time coincided with the overall progression of competitive swimming performance during the most recent Olympic Games.

### Collective Interpretation of the Olympic Cycles Associated with the Largest Performance Improvements

#### 1956 – The Onset of Modern Olympic Pools and the First Wave of Technological Advancements.

The 1956 Melbourne Olympic Games coincided with one of the earliest periods associated with substantial performance improvements in Olympic swimming. This was the first time competitions were held in a fully enclosed and environmentally controlled Olympic swimming and diving pool, which brought major improvements in wave-dampening gutter systems, deeper and wider pool basins, stabilized water temperature and clarity, insulation from outdoor environmental disturbances [36]. Together, these developments coincided with measurable reductions in race times across multiple swimming strokes. In parallel, the emergence of synthetic swimsuit materials (e.g., nylon-based fabrics) reduced form drag and enhanced body alignment in the water which may have been particularly relevant for high-drag strokes such as breaststroke and butterfly [37]. Taken together, the 1956 Olympic Games were associated with one of the earliest periods in which substantial performance improvements were observed across multiple swimming strokes, coinciding with developments in pool engineering, competition environments, and early swimsuit technology.

#### 1972 – Technical Standardization, Optimized Pool Engineering, and the Maturation of Equipment Use.

The 1972 Munich Games were associated with one of the largest percentage improvements in swimming performance observed in the present study, coinciding with advances in biomechanical understanding and pool engineering [2]. During this era, improvements in pool engineering — especially the adoption of anti-wave lane ropes and deeper, more controlled “fast-pool” designs with advanced water circulation and gutter systems — were associated with reduced turbulence and lane-to-lane interference, which may have supported performance improvements among technically proficient swimmers [38]. Simultaneously, the 1970s marked the rise of modern training science—including structured interval training, lactate-guided pacing, and improved aerobic conditioning methods [39–42]. These developments collectively improved stroke efficiency across all styles: (a) higher tempo control in freestyle, (b) improved body alignment in backstroke, (c) reduced velocity fluctuations in breaststroke, (d) more efficient undulation rhythm in butterfly. Taken together, the 1972 Olympic Games coincided with a period in which some of the largest percentage improvements in swimming performance were observed, alongside developments in technical refinement, pool design, and sports-science practices.

#### 2008 – Polyurethane Swimsuits and the Technological Performance Revolution.

The 2008 Beijing Olympic Games were associated with one of the largest percentage improvements in swimming performance observed in the present study, coinciding with the widespread adoption of polyurethane-based full-body racing suits (e.g., the LZR Racer) [25,31,43]. These suits — particularly polyurethane-based full-body models such as Speedo’s LZR Racer — compressed the swimmer’s body into a more hydrodynamic form, lowered passive drag and turbulence, and improved buoyancy and body-line stability through tight compression panels and ultra-smooth external textures [43]. Resulting performance improvements of approximately 1.5–3.5% were reported in elite competitive settings [25,43,44]. Additionally, this period coincided with upgrades in starting block design, improved water-flow systems in competition pools, and sophisticated underwater race analysis. Taken together, the 2008 Olympic Games coincided with a period in which substantial performance improvements were observed across multiple swimming strokes, alongside developments in swimsuit technology, pool systems, and race-analysis methods.

Several limitations of the present study should be acknowledged. First, performance analyses were based on mean finalist values rather than winning performances. Although this approach provides a broader representation of elite competitive standards within each Olympic Games, it may have attenuated some peak-performance differences that could be observed when focusing exclusively on Olympic champions. Second, although major technological, regulatory, and methodological developments occurred throughout the study period, these factors were not directly incorporated into the statistical analyses. Consequently, the present findings should be interpreted as descriptive of long-term performance evolution rather than as evidence of the isolated effects of specific historical developments. Third, although performance changes were evaluated across successive Olympic Games, formal trend-modeling or change-point analyses were not performed. Therefore, the Olympic cycles associated with the largest percentage improvements should be interpreted descriptively rather than as statistically verified transition points. Future studies employing segmented regression or change-point approaches may provide additional insight into the temporal dynamics of Olympic swimming performance. Fourth, reaction-time analyses were limited to Olympic Games for which reaction-time data were publicly available. Therefore, long-term comparisons of reaction-time evolution should be interpreted within the context of the available historical records rather than across the entire 1924–2024 study period.

## Conclusion

This century-long analysis provides a comprehensive overview of changes in Olympic swimming performance between 1924 and 2024. The largest percentage improvements were observed during specific Olympic Games, including 1956, 1972, and 2008, which coincided with periods of substantial technological, regulatory, and methodological developments in competitive swimming. Across all strokes and distances, swimming speeds increased while race times and reaction times generally decreased over time. Collectively, these findings illustrate the substantial long-term progression of Olympic swimming performance and provide valuable historical benchmarks for coaches, athletes, and sports scientists. Understanding these historical trajectories may support future performance forecasting and evidence-informed decision making in coaching, talent development, and sport technology design.

## Supporting information

S1 File S1–S9 Tables. Percentage changes in final performance times, swimming speeds, and reaction times across consecutive available Olympic Games.
(DOCX)

S2 File Olympic_Swimming_Minimal_Dataset_1924_2024. (XLSX)

## References

[1] World Aquatics. Latest competition results. https://www.worldaquatics.com/swimming/results?year=2025&month=latest&disciplines=SW. 2025. Accessed 2025 December 25.

[2] Hołub M, Stanula A, Baron J, Głyk W, Rosemann T, Knechtle B. Predicting breaststroke and butterfly stroke results in swimming based on olympics history. Int J Environ Res Public Health. 2021;18(12):6621. doi: 10.3390/ijerph18126621 34202950 PMC8296408

[3] Barbosa TM, Fernandes R, Keskinen KL, Colaço P, Cardoso C, Silva J, et al. Evaluation of the energy expenditure in competitive swimming strokes. Int J Sports Med. 2006;27(11):894–9. doi: 10.1055/s-2006-923776 16612740

[4] Barbosa TM, Bragada JA, Reis VM, Marinho DA, Carvalho C, Silva AJ. Energetics and biomechanics as determining factors of swimming performance: updating the state of the art. J Sci Med Sport. 2010;13(2):262–9. doi: 10.1016/j.jsams.2009.01.003 19409842

[5] Pelayo P, Alberty M. The history of swimming research. In: Seifert L, Chollet D, Mujika I, editors. World Book of Swimming: From Science to Performance. New York: Nova Science Publishers. 2011. p. 19–26.

[6] Trendafilov D. Development of style breast stroke from the antiquity to 1952. Acta Physiol Educ Sport. 2015;5:231–3.

[7] World Aquatics. Swimming rules. https://www.worldaquatics.com/swimming/rules. 2025. Accessed 2025 December 25.

[8] Stanula A, Maszczyk A, Roczniok R, Pietraszewski P, Ostrowski A, Zając A, et al. The development and prediction of athletic performance in freestyle swimming. J Hum Kinet. 2012;32:97–107. doi: 10.2478/v10078-012-0027-3 23486223 PMC3590874

[9] Dustker SM, Nayak DM. Performance analysis of individual swimmers for elite competitive swimming. Int J Eng Res Adv Technol. 2016;2:142–52.

[10] O’connor LM, Vozenilek JA. Is it the athlete or the equipment? An analysis of the top swim performances from 1990 to 2010. J Strength Cond Res. 2011;25(12):3239–41. doi: 10.1519/JSC.0b013e3182392c5f 21964430

[11] Lippi G, Banfi G, Favaloro EJ, Rittweger J, Maffulli N. Updates on improvement of human athletic performance: focus on world records in athletics. Br Med Bull. 2008;87:7–15. doi: 10.1093/bmb/ldn029 18723588

[12] Gonjo T, Olstad BH. Race analysis in competitive swimming: A narrative review. Int J Environ Res Public Health. 2020;18(1):69. doi: 10.3390/ijerph18010069 33374118 PMC7795652

[13] Costa MJ, Garrido ND, Marinho DA, Santos CC. How Much the Swimming Performance Leading to Tokyo 2020 Olympic Games Was Impaired Due to the Covid-19 Lockdown?. J Sports Sci Med. 2021;20(4):714–20. doi: 10.52082/jssm.2021.714 35321138 PMC8488832

[14] Knechtle B, Bragazzi NL, König S, Nikolaidis PT, Wild S, Rosemann T. The age in swimming of champions in World Championships (1994–2013) and Olympic Games (1992–2012): A cross-sectional data analysis. Sports. 2016;4:1–27. doi: 10.3390/sports4010017 PMC5968937 29910265

[15] Fang J, Li Y, Cheng Y. The variability of competitive performance and pacing strategies in different rounds of the 400 m and 800 m freestyle swimming races at the 2017-2024 World Swimming Championships. Front Sports Act Living. 2024;6:1496878. doi: 10.3389/fspor.2024.1496878 39539312 PMC11557356

[16] da Silva JKF, Enes AAN, Sotomaior BB, Ruy Barbosa MA, de Souza RO, Osiecki R. Analysis of the performance of finalist swimming athletes in Olympic games: Reaction time, partial time, speed, and final time. J Phys Educ Sport. 2020;20:539–45. doi: 10.7752/jpes.2020.02080

[17] Cengiz ŞŞ, Coşkun EŞ. Swimming in the Olympics. Int J Sport Cult Sci. 2023;11:56–70.

[18] Veiga S, Grenouillat A, Rodríguez-Adalia L, Zarzosa-Alonso F, Pla R. Ten-Year Evolution of World Swimming Trends for Different Performance Clusters: A Gaussian Model. Int J Sports Physiol Perform. 2024;19(12):1391–9. doi: 10.1123/ijspp.2024-0206 39348884

[19] Costa MJ, Marinho DA, Reis VM, Silva AJ, Marques MC, Bragada JA, et al. Tracking the performance of world-ranked swimmers. J Sports Sci Med. 2010;9(3):411–7. 24149635 PMC3761712

[20] Colwin C. Looking back, looking ahead. Breakthrough Swimming. Champaign, IL, USA: Human Kinetics. 2002. p. 217–28.

[21] Colwin CM, Colwin CM. Swimming into the 21st century (Vol. 1). Champaign: Leisure Press; 1992.

[22] Marinho DA, Willemsen D, Barbosa TM, Silva AJ, Vilas-Boas JP, Neiva HP, et al. Numerical simulations of a swimmer’s head and cap wearing different types of goggles. Sports Biomech. 2024;23(9):1123–35. doi: 10.1080/14763141.2021.1923793 34080520

[23] Chiswell B, Wildsoet CF. On the possible sources of ocular irritation in chlorinated swimming pools. Clin Exp Optom. 1986;69:178–82.

[24] Berthelot G, Len S, Hellard P, Tafflet M, El Helou N, Escolano S, et al. Technology & swimming: 3 steps beyond physiology. Materials Today. 2010;13(11):46–51. doi: 10.1016/s1369-7021(10)70203-0

[25] Morales AT, Tamayo Fajardo JA, González-García H. High-Speed Swimsuits and Their Historical Development in Competitive Swimming. Front Psychol. 2019;10:2639. doi: 10.3389/fpsyg.2019.02639 31920770 PMC6927279

[26] Phillips CWG, Forrester AIJ, Hudson DA, Turnock SR. Propulsive efficiency of alternative underwater flykick techniques for swimmers. Proc Inst Mech Eng Part P J Sport Eng Technol. 2021;235:354–64. doi: 10.1177/1754337120912610

[27] Kardjono K, Rachmawati M. The relation between underwater length and the swimming speed of 50 meters backstroke. Adv Heal Sci Res. 2019;11:250–2.

[28] World A. World aquatics competition regulations (section 16.1.7.1 — lane ropes and wave reduction). 2023. https://resources.fina.org/fina/document/2023/04/05/c8f2e9bf-54bb-4e95-a534-116671049357/WORLD_AQUATICS_COMPETITION_REGULATIONS.pdf

[29] Rizk NP. Wave-damping properties of swimming lines. Lund: Lund University. 2014. https://lup.lub.lu.se/student-papers/record/4406509

[30] Issurin V, Pushkar-Verbitsky V, Verbitsky O. Effect of high-tech swimsuits on the swimming performance in top-level swimmers. J Sports Med Phys Fitness. 2014;54(4):383–8. 24598554

[31] Palma DD, Ascione A. New technologies in swimming sports. Ital J Heal Educ Sport Incl Didact. 2018;2:41–53.

[32] Costa MJ, Quinta-Nova L, Ferreira S, Costa AM, Santos CC. Trend Forecasting in Swimming World Records and in the Age of World Record Holders. Applied Sciences. 2024;14(20):9492. doi: 10.3390/app14209492

[33] Barney DE, Barney RK. A long night’s journey into day: The Odyssey of butterfly. J Olympic Hist. 2008;16:11–25.

[34] Yamakawa KK, Takagi H, Sengoku Y. In: Tokyo, Japan, 2018. 187–92.

[35] Wądrzyk Ł, Nosiadek L, Staszkiewicz R. Underwater dolphin kicks of young swimmers–evaluation of effectiveness based on kinematic analysis. Hum Mov. 2017;18:23–9. doi: 10.1515/humo-2017-0030

[36] Davison G. Welcoming the world: The 1956 Olympic Games and the re‐presentation of Melbourne. Aust Hist Stud. 1997;27:64–76. doi: 10.1080/10314619708596043

[37] Stefani R. Olympic Swimming Gold: The Suit or the Swimmer in the Suit?. Significance. 2012;9(2):13–7. doi: 10.1111/j.1740-9713.2012.00553.x

[38] Anti Wave International. Anti wave racing lanes - since Munich 1972. https://www.anti.to/wp-content/uploads/2021/01/AntiWaveRacingLanes2020web.pdf. 2000.

[39] Maglischo EW. Swimming Fastest. Champaign, IL: Human Kinetics. 2003.

[40] Olbrecht J. Lactate production and metabolism in swimming. In: Seifert L, Chollet D, Mujika I, editors. World Book of Swimming: From Science to Performance. New York: Nova. 2010. p. 255–76.

[41] Boulgakova NZ, Popov OI, Issurin VB. Competitive swimming. EOLSS Publishers. 2012.

[42] Hawley JA. State of the art training guidelines for endurance performance. South African J Sport Med. 1995;2:7–12.

[43] Foster L, James D, Haake S. Influence of full body swimsuits on competitive performance. Procedia Engineering. 2012;34:712–7. doi: 10.1016/j.proeng.2012.04.121

[44] Allen SV, Vandenbogaerde TJ, Hopkins WG. Career performance trajectories of Olympic swimmers: benchmarks for talent development. Eur J Sport Sci. 2014;14(7):643–51. doi: 10.1080/17461391.2014.893020 24597644

