# Supplementary material for: Performance evolution in Olympic swimming over the last 100 years: A retrospective stroke-based analysis
Source: PLoS One. 2026 Oct 26;21(10):e0361289. doi: 10.1371/journal.pone.0361289 (PMC13704801; doi:10.1371/journal.pone.0361289)
Supplement: Supplementary file 1 [file pone.0361289.s001.docx]

**S1 Table. Percentage changes in final performance times for the 50-, 100-, and 200-m freestyle events across consecutive Olympic Games (1924–2024)**

|  | **50m** | **50m** | **100m** | **100m** | **200m** | **200m** |
| --- | --- | --- | --- | --- | --- | --- |
|  | **F** | **M** | **F** | **M** | **F** | **M** |
| 1924 | NA | NA | NA | NA | NA | NA |
| 1928 | NA | NA | 2.5% | 1.6% | NA | NA |
| 1932 | NA | NA | 6.1% | 2.6% | NA | NA |
| 1936 | NA | NA | 2.0% | 0.5% | NA | NA |
| 1948 | NA | NA | -0.8% | 0.0% | NA | NA |
| 1952 | NA | NA | 0.8% | 0.6% | NA | NA |
| 1956 | NA | NA | 4.1% | 2.1% | NA | NA |
| 1960 | NA | NA | 1.6% | 2.0% | NA | NA |
| 1964 | NA | NA | 3.3% | 2.9% | NA | NA |
| 1968 | NA | NA | 1.1% | 1.8% | NA | NA |
| 1972 | NA | NA | 2.2% | 2.2% | 4.7% | 2.9% |
| 1976 | NA | NA | 4.6% | 1.4% | 3.1% | 3.2% |
| 1980 | NA | NA | -0.1% | -0.1% | 0.7% | -0.5% |
| 1984 | NA | NA | 0.2% | 2.0% | 1.1% | 1.3% |
| 1988 | NA | NA | 1.6% | 1.3% | 0.7% | 2.0% |
| 1992 | 1.6% | 1.5% | 0.6% | 0.4% | -0.2% | 0.2% |
| 1996 | 0.0% | -0.4% | 0.2% | 0.6% | 0.0% | -0.2% |
| 2000 | 1.4% | 1.2% | 1.1% | 0.7% | 0.9% | 0.9% |
| 2004 | 0.2% | 0.4% | 0.3% | 0.3% | 0.2% | 0.9% |
| 2008 | 2.4% | 2.4% | 1.4% | 2.1% | 2.0% | 0.6% |
| 2012 | -0.4% | -0.5% | 0.6% | -0.2% | 0.1% | 0.2% |
| 2016 | 0.9% | 0.0% | 0.8% | -0.2% | 1.0% | 0.1% |
| 2020 | 0.0% | 0.3% | 0.8% | 0.7% | 0.1% | 0.6% |
| 2024 | 0.2% | 0.4% | 0.1% | 0.2% | 0.0% | -0.3% |
| **Total percentage change** | **6.1%** | **5.4%** | **30.0%** | **22.6%** | **13.6%** | **11.2%** |

Note. Percentage change in final performance time was calculated as [(previous available Olympic mean − current Olympic mean) / previous available Olympic mean] × 100, using mean values only; standard deviations were not included. Positive values indicate a decrease in final time, whereas negative values indicate an increase. Total percentage change was calculated between the first available Olympic mean and the 2024 Olympic mean using the same formula. F: Female; M: Male; NA: Not available/not applicable because no previous comparable Olympic value was available.

**S2 Table. Percentage changes in final performance times for the 400-, 800-, and 1500-m freestyle events across consecutive Olympic Games (1924–2024)**

|  | **400m** | **400m** | **800m** | **800m** | **1500m** | **1500m** |
| --- | --- | --- | --- | --- | --- | --- |
|  | **F** | **M** | **F** | **M** | **F** | **M** |
| 1924 | NA | NA | NA | NA | NA | NA |
| 1928 | 2.2% | 1.7% | NA | NA | NA | 2.7% |
| 1932 | 3.0% | 5.4% | NA | NA | NA | 5.3% |
| 1936 | 4.5% | 0.5% | NA | NA | NA | -0.2% |
| 1948 | 2.7% | 0.6% | NA | NA | NA | -1.4% |
| 1952 | 2.3% | 4.3% | NA | NA | NA | 5.0% |
| 1956 | 2.3% | 1.1% | NA | NA | NA | 2.9% |
| 1960 | 4.2% | 3.9% | NA | NA | NA | 4.0% |
| 1964 | 3.0% | 2.4% | NA | NA | NA | 2.4% |
| 1968 | 2.7% | 0.4% | NA | NA | NA | -0.2% |
| 1972 | 5.9% | 4.7% | 6.1% | NA | NA | 6.2% |
| 1976 | 3.6% | 2.9% | 3.2% | NA | NA | 5.5% |
| 1980 | 0.7% | 0.9% | 1.2% | NA | NA | 0.4% |
| 1984 | 0.1% | 0.6% | 0.1% | NA | NA | -0.4% |
| 1988 | 1.5% | 2.1% | 1.9% | NA | NA | 0.9% |
| 1992 | -0.9% | 0.4% | -1.2% | NA | NA | 0.5% |
| 1996 | 0.6% | -1.2% | 0.0% | NA | NA | -0.2% |
| 2000 | 0.2% | 1.9% | 1.4% | NA | NA | 0.9% |
| 2004 | 0.6% | 0.1% | -0.2% | NA | NA | 0.4% |
| 2008 | 1.3% | 1.0% | 1.0% | NA | NA | 1.0% |
| 2012 | 0.3% | -0.8% | 0.2% | NA | NA | 0.2% |
| 2016 | 0.4% | 0.6% | 0.8% | NA | NA | 0.1% |
| 2020 | 0.2% | 0.0% | -0.2% | NA | NA | -0.4% |
| 2024 | 0.6% | 0.3% | 0.6% | 0.6% | -0.1% | 1.1% |
| **Total percentage change** | **34.6%** | **29.0%** | **14.0%** | **0.6%** | **-0.1%** | **31.2%** |

Note. Percentage change in final performance time was calculated as [(previous available Olympic mean − current Olympic mean) / previous available Olympic mean] × 100, using mean values only; standard deviations were not included. Positive values indicate a decrease in final time, whereas negative values indicate an increase. Total percentage change was calculated between the first available Olympic mean and the 2024 Olympic mean using the same formula. F: Female; M: Male; NA: Not available/not applicable because no previous comparable Olympic value was available.

**S3 Table. Percentage changes in final performance swimming speeds for the 50-, 100-, and 200-m freestyle events across consecutive Olympic Games (1924–2024)**

|  | **50m** | **50m** | **100m** | **100m** | **200m** | **200m** |
| --- | --- | --- | --- | --- | --- | --- |
|  | **F** | **M** | **F** | **M** | **F** | **M** |
| 1924 | NA | NA | NA | NA | NA | NA |
| 1928 | NA | NA | 2.4% | 1.7% | NA | NA |
| 1932 | NA | NA | 6.5% | 2.5% | NA | NA |
| 1936 | NA | NA | 2.1% | 0.5% | NA | NA |
| 1948 | NA | NA | -0.7% | 0.0% | NA | NA |
| 1952 | NA | NA | 0.7% | 0.6% | NA | NA |
| 1956 | NA | NA | 4.3% | 2.2% | NA | NA |
| 1960 | NA | NA | 1.7% | 2.0% | NA | NA |
| 1964 | NA | NA | 3.4% | 3.0% | NA | NA |
| 1968 | NA | NA | 1.2% | 1.8% | NA | NA |
| 1972 | NA | NA | 2.2% | 2.3% | 5.0% | 3.0% |
| 1976 | NA | NA | 4.8% | 1.5% | 3.2% | 3.3% |
| 1980 | NA | NA | -0.1% | -0.1% | 0.7% | -0.5% |
| 1984 | NA | NA | 0.2% | 2.0% | 1.2% | 1.3% |
| 1988 | NA | NA | 1.6% | 1.4% | 0.7% | 2.0% |
| 1992 | 1.6% | 1.6% | 0.6% | 0.4% | -0.2% | 0.2% |
| 1996 | 0.0% | -0.4% | 0.2% | 0.5% | 0.0% | -0.2% |
| 2000 | 1.4% | 1.3% | 1.2% | 0.7% | 0.8% | 0.9% |
| 2004 | 0.2% | 0.3% | 0.3% | 0.3% | 0.2% | 0.9% |
| 2008 | 2.5% | 2.5% | 1.5% | 2.1% | 2.0% | 0.7% |
| 2012 | -0.4% | -0.5% | 0.5% | -0.2% | 0.1% | 0.2% |
| 2016 | 0.9% | 0.0% | 0.9% | -0.2% | 1.0% | 0.1% |
| 2020 | 0.0% | 0.3% | 0.8% | 0.7% | 0.1% | 0.6% |
| 2024 | 0.1% | 0.4% | 0.1% | 0.2% | 0.0% | -0.3% |
| **Total percentage change** | **6.5%** | **5.7%** | **42.7%** | **29.2%** | **15.6%** | **12.6%** |

Note. Percentage change in swimming speed was calculated as [(current Olympic mean − previous available Olympic mean) / previous available Olympic mean] × 100, using mean values only; standard deviations were not included. Positive values indicate an increase in swimming speed, whereas negative values indicate a decrease. Total percentage change was calculated between the first available Olympic mean and the 2024 Olympic mean using the same formula. F: Female; M: Male; NA: Not available/not applicable because no previous comparable Olympic value was available.

**S4 Table. Percentage changes in final performance swimming speeds for the 400-, 800-, and 1500-m freestyle events across consecutive Olympic Games (1924–2024)**

|  | **400m** | **400m** | **800m** | **800m** | **1500m** | **1500m** |
| --- | --- | --- | --- | --- | --- | --- |
|  | **F** | **M** | **F** | **M** | **F** | **M** |
| 1924 | NA | NA | NA | NA | NA | NA |
| 1928 | 2.1% | 1.7% | NA | NA | NA | 2.7% |
| 1932 | 3.3% | 5.6% | NA | NA | NA | 5.5% |
| 1936 | 4.5% | 0.5% | NA | NA | NA | -0.2% |
| 1948 | 2.7% | 0.6% | NA | NA | NA | -1.4% |
| 1952 | 2.4% | 4.4% | NA | NA | NA | 5.3% |
| 1956 | 2.5% | 1.2% | NA | NA | NA | 3.0% |
| 1960 | 4.3% | 4.0% | NA | NA | NA | 4.1% |
| 1964 | 3.1% | 2.5% | NA | NA | NA | 2.4% |
| 1968 | 2.7% | 0.4% | NA | NA | NA | -0.1% |
| 1972 | 6.2% | 4.9% | 6.5% | NA | NA | 6.5% |
| 1976 | 3.8% | 3.0% | 3.3% | NA | NA | 5.8% |
| 1980 | 0.7% | 0.9% | 1.2% | NA | NA | 0.4% |
| 1984 | 0.1% | 0.6% | 0.1% | NA | NA | -0.4% |
| 1988 | 1.6% | 2.1% | 1.9% | NA | NA | 0.9% |
| 1992 | -1.0% | 0.4% | -1.2% | NA | NA | 0.5% |
| 1996 | 0.6% | -1.2% | 0.0% | NA | NA | -0.2% |
| 2000 | 0.3% | 1.9% | 1.4% | NA | NA | 1.0% |
| 2004 | 0.6% | 0.2% | -0.3% | NA | NA | 0.4% |
| 2008 | 1.3% | 1.0% | 1.0% | NA | NA | 1.9% |
| 2012 | 0.2% | -0.8% | 0.3% | NA | NA | -0.8% |
| 2016 | 0.5% | 0.6% | 0.9% | NA | NA | 0.1% |
| 2020 | 0.2% | -0.1% | -0.3% | NA | NA | 0.0% |
| 2024 | 0.5% | 0.3% | 0.6% | 0.5% | -0.1% | 0.7% |
| **Total percentage change** | **52.7%** | **40.6%** | **16.2%** | **0.5%** | **-0.1%** | **45.2%** |

Note. Percentage change in swimming speed was calculated as [(current Olympic mean − previous available Olympic mean) / previous available Olympic mean] × 100, using mean values only; standard deviations were not included. Positive values indicate an increase in swimming speed, whereas negative values indicate a decrease. Total percentage change was calculated between the first available Olympic mean and the 2024 Olympic mean using the same formula. F: Female; M: Male; NA: Not available/not applicable because no previous comparable Olympic value was available.

**S5 Table. Percentage changes in reaction times for freestyle final performances across consecutive Olympic Games (2000–2024)**

|  | **50m** | **50m** | **100m** | **100m** | **200m** | **200m** |
| --- | --- | --- | --- | --- | --- | --- |
|  | **F** | **M** | **F** | **M** | **F** | **M** |
| 2000 | NA | NA | NA | NA | NA | NA |
| 2004 | 0.0% | 12.7% | -1.4% | 6.3% | 1.2% | 1.3% |
| 2008 | -1.3% | -1.4% | -4.0% | 4.1% | 7.1% | 7.6% |
| 2012 | 6.6% | NA | 6.4% | 2.8% | 7.7% | 4.1% |
| 2016 | 4.2% | 5.7% | 4.1% | 2.9% | 1.4% | 0.0% |
| 2020 | 1.5% | 4.5% | 4.3% | 6.0% | 2.8% | 5.7% |
| 2024 | -1.5% | 1.6% | -1.5% | -3.2% | -1.4% | -3.0% |
| **Total percentage change** | **9.3%** | **21.5%** | **8.1%** | **17.7%** | **17.6%** | **15.0%** |
|  | **400m** | **400m** | **800m** | **800m** | **1500m** | **1500m** |
|  | **F** | **M** | **F** | **M** | **F** | **M** |
| 2000 | NA | NA | NA | NA | NA | NA |
| 2004 | -1.2% | -1.2% | -1.2% | NA | NA | 1.2% |
| 2008 | 5.8% | 8.4% | 5.8% | NA | NA | 2.4% |
| 2012 | 9.9% | 6.6% | 9.9% | NA | NA | 2.5% |
| 2016 | 1.4% | -1.4% | 0.0% | NA | NA | 6.3% |
| 2020 | 0.0% | 1.4% | 1.4% | NA | NA | 1.4% |
| 2024 | -1.4% | 5.6% | -1.4% | 2.7% | -1.4% | -1.4% |
| **Total percentage change** | **14.1%** | **18.3%** | **14.1%** | **2.7%** | **-1.4%** | **11.9%** |

Note. Percentage change in reaction time was calculated as [(previous available Olympic mean − current Olympic mean) / previous available Olympic mean] × 100, using mean values only; standard deviations were not included. Positive values indicate a decrease in reaction time, whereas negative values indicate an increase. Total percentage change was calculated between the first available Olympic mean and the 2024 Olympic mean using the same formula. F: Female; M: Male; NA: Not available/not applicable because no previous comparable Olympic value was available.

**S6 Table. Percentage changes in backstroke final performance times and swimming speeds across consecutive Olympic Games (1924–2024)**

|  | **Final time** | **Final time** | **Final time** | **Final time** | **Swimming speed** | **Swimming speed** | **Swimming speed** | **Swimming speed** |
| --- | --- | --- | --- | --- | --- | --- | --- | --- |
|  | **100m** | **100m** | **200m** | **200m** | **100m** | **100m** | **200m** | **200m** |
|  | **F** | **M** | **F** | **M** | **F** | **M** | **F** | **M** |
| 1924 | NA | NA | NA | NA | NA | NA | NA | NA |
| 1928 | 5.1% | 8.8% | NA | NA | 5.2% | 9.3% | NA | NA |
| 1932 | 1.9% | 2.2% | NA | NA | 2.0% | 2.2% | NA | NA |
| 1936 | 2.6% | 2.4% | NA | NA | 2.6% | 2.4% | NA | NA |
| 1948 | 2.9% | 0.8% | NA | NA | 3.0% | 0.8% | NA | NA |
| 1952 | 2.0% | 1.7% | NA | NA | 2.0% | 1.7% | NA | NA |
| 1956 | 3.2% | 3.6% | NA | NA | 3.3% | 3.8% | NA | NA |
| 1960 | 3.2% | 2.5% | NA | NA | 3.3% | 2.5% | NA | NA |
| 1964 | 3.3% | NA | NA | NA | 3.4% | NA | NA | NA |
| 1968 | 0.8% | 3.5% | NA | NA | 0.9% | 3.6% | NA | NA |
| 1972 | 2.6% | 3.5% | 5.2% | 4.6% | 2.6% | 3.6% | 5.5% | 4.8% |
| 1976 | 3.6% | 2.7% | 4.6% | 2.6% | 3.8% | 2.8% | 4.9% | 2.7% |
| 1980 | 1.4% | -0.7% | 0.8% | 0.0% | 1.5% | -0.7% | 0.9% | -0.1% |
| 1984 | -4.8% | 1.2% | 0.3% | 0.6% | -0.1% | 1.2% | 0.2% | 0.6% |
| 1988 | 5.6% | 2.2% | 1.4% | 1.6% | 1.1% | 2.3% | 1.5% | 1.6% |
| 1992 | 1.3% | 1.0% | 1.3% | 1.2% | 1.3% | 1.0% | 1.3% | 1.1% |
| 1996 | -0.6% | -0.1% | -0.9% | -1.1% | -0.6% | -0.1% | -0.9% | -1.0% |
| 2000 | 1.7% | 0.6% | 0.5% | 2.0% | 1.7% | 0.6% | 0.5% | 2.1% |
| 2004 | 0.1% | 0.6% | 0.8% | 0.4% | 0.1% | 0.6% | 0.7% | 0.4% |
| 2008 | 2.5% | 2.3% | 2.2% | 2.1% | 2.6% | 2.3% | 2.3% | 2.1% |
| 2012 | 0.7% | 0.2% | 0.4% | -0.1% | 0.7% | 0.2% | 0.4% | -0.1% |
| 2016 | 0.5% | 0.9% | -0.5% | 0.5% | 0.5% | 0.9% | -0.4% | 0.5% |
| 2020 | 0.7% | 0.5% | 0.9% | -0.6% | 0.8% | 0.5% | 0.8% | -0.6% |
| 2024 | -0.3% | -0.1% | 0.1% | 0.5% | -0.3% | -0.1% | 0.1% | 0.5% |
| **Total percentage change** | **33.5%** | **33.7%** | **16.1%** | **13.4%** | **50.3%** | **50.1%** | **19.2%** | **15.4%** |

Note. For final performance time, percentage change was calculated as [(previous available Olympic mean − current Olympic mean) / previous available Olympic mean] × 100. For swimming speed, percentage change was calculated as [(current Olympic mean − previous available Olympic mean) / previous available Olympic mean] × 100. Mean values only were used; standard deviations were not included. Thus, positive values indicate either a decrease in final time or an increase in swimming speed. Total percentage change was calculated between the first available Olympic mean and the 2024 Olympic mean using the corresponding formula. F: Female; M: Male; NA: Not available/not applicable because no previous comparable Olympic value was available.

**S7 Table. Percentage changes in breaststroke final performance times and swimming speeds across consecutive Olympic Games (1924–2024)**

|  | **Final time** | **Final time** | **Final time** | **Final time** | **Swimming speed** | **Swimming speed** | **Swimming speed** | **Swimming speed** |
| --- | --- | --- | --- | --- | --- | --- | --- | --- |
|  | **100m** | **100m** | **200m** | **200m** | **100m** | **100m** | **200m** | **200m** |
|  | **F** | **M** | **F** | **M** | **F** | **M** | **F** | **M** |
| 1924 | NA | NA | NA | NA | NA | NA | NA | NA |
| 1928 | NA | NA | 9.2% | 3.6% | NA | NA | 10.2% | 3.7% |
| 1932 | NA | NA | 3.4% | 3.6% | NA | NA | 3.5% | 3.7% |
| 1936 | NA | NA | 1.5% | 1.4% | NA | NA | 1.5% | 1.5% |
| 1948 | NA | NA | 3.0% | 0.7% | NA | NA | 2.7% | 0.7% |
| 1952 | NA | NA | 2.7% | 4.3% | NA | NA | 3.2% | 4.4% |
| 1956 | NA | NA | 0.9% | -0.5% | NA | NA | 0.9% | -0.5% |
| 1960 | NA | NA | 1.3% | -1.0% | NA | NA | 1.3% | -0.9% |
| 1964 | NA | NA | 2.3% | 5.0% | NA | NA | 2.3% | 5.3% |
| 1968 | NA | NA | 0.9% | 0.1% | NA | NA | 0.9% | 0.2% |
| 1972 | 0.6% | 3.5% | 3.0% | 4.3% | 0.6% | 3.6% | 3.0% | 4.4% |
| 1976 | 3.5% | 2.8% | 3.9% | 3.7% | 3.6% | 2.9% | 4.0% | 3.8% |
| 1980 | 2.7% | -0.3% | 2.6% | 0.4% | 2.7% | -0.3% | 2.7% | 0.4% |
| 1984 | 0.7% | 2.0% | -0.2% | 1.2% | 0.7% | 2.1% | -0.2% | 1.2% |
| 1988 | 2.4% | 0.9% | 2.9% | 1.4% | 2.4% | 0.9% | 2.9% | 1.4% |
| 1992 | -0.4% | 0.9% | -0.3% | 2.1% | -0.3% | 0.9% | -0.2% | 2.2% |
| 1996 | 0.8% | 0.7% | 0.8% | -1.1% | 0.8% | 0.7% | 0.8% | -1.1% |
| 2000 | 1.2% | 0.3% | 1.4% | 1.1% | 1.2% | 0.4% | 1.4% | 1.1% |
| 2004 | 1.1% | 0.2% | 0.1% | 1.3% | 1.2% | 0.2% | 0.1% | 1.3% |
| 2008 | 0.1% | 2.5% | 1.5% | 1.3% | 0.1% | 2.5% | 1.5% | 1.3% |
| 2012 | 1.0% | 0.1% | 0.6% | 0.7% | 1.1% | 0.1% | 0.6% | 0.7% |
| 2016 | 0.2% | 1.0% | 0.0% | 0.6% | 0.2% | 1.1% | 0.0% | 0.6% |
| 2020 | 0.9% | 0.6% | 0.5% | 0.1% | 0.9% | 0.6% | 0.5% | 0.1% |
| 2024 | 0.1% | -1.2% | -0.6% | -0.6% | 0.0% | -1.2% | -0.6% | -0.6% |
| **Total percentage change** | **14.0%** | **13.3%** | **34.5%** | **29.0%** | **16.2%** | **15.4%** | **52.8%** | **40.8%** |

Note. For final performance time, percentage change was calculated as [(previous available Olympic mean − current Olympic mean) / previous available Olympic mean] × 100. For swimming speed, percentage change was calculated as [(current Olympic mean − previous available Olympic mean) / previous available Olympic mean] × 100. Mean values only were used; standard deviations were not included. Thus, positive values indicate either a decrease in final time or an increase in swimming speed. Total percentage change was calculated between the first available Olympic mean and the 2024 Olympic mean using the corresponding formula. F: Female; M: Male; NA: Not available/not applicable because no previous comparable Olympic value was available.

**S8 Table. Percentage changes in butterfly final performance times and swimming speeds across consecutive Olympic Games (1956–2024)**

|  | **Final time** | **Final time** | **Final time** | **Final time** | **Swimming speed** | **Swimming speed** | **Swimming speed** | **Swimming speed** |
| --- | --- | --- | --- | --- | --- | --- | --- | --- |
|  | **100m** | **100m** | **200m** | **200m** | **100m** | **100m** | **200m** | **200m** |
|  | **F** | **M** | **F** | **M** | **F** | **M** | **F** | **M** |
| 1924 | NA | NA | NA | NA | NA | NA | NA | NA |
| 1928 | NA | NA | NA | NA | NA | NA | NA | NA |
| 1932 | NA | NA | NA | NA | NA | NA | NA | NA |
| 1936 | NA | NA | NA | NA | NA | NA | NA | NA |
| 1948 | NA | NA | NA | NA | NA | NA | NA | NA |
| 1952 | NA | NA | NA | NA | NA | NA | NA | NA |
| 1956 | NA | NA | NA | NA | NA | NA | NA | NA |
| 1960 | 3.9% | NA | NA | 6.4% | 4.0% | NA | NA | 6.8% |
| 1964 | 6.7% | NA | NA | 4.4% | 7.2% | NA | NA | 4.6% |
| 1968 | 1.2% | NA | NA | 0.0% | 1.2% | NA | NA | 0.0% |
| 1972 | 4.0% | 2.9% | 6.6% | 5.2% | 4.1% | 3.0% | 7.0% | 5.5% |
| 1976 | 3.8% | 1.7% | 4.0% | 2.4% | 4.0% | 1.7% | 4.2% | 2.4% |
| 1980 | -0.8% | 0.0% | 0.1% | -0.8% | -0.8% | -0.1% | 0.1% | -0.8% |
| 1984 | 1.2% | 2.4% | 0.5% | 2.6% | 1.2% | 2.5% | 0.6% | 2.7% |
| 1988 | 1.8% | 0.7% | 0.8% | -0.2% | 1.8% | 0.6% | 0.8% | -0.2% |
| 1992 | 0.8% | -0.4% | 0.6% | 0.3% | 0.8% | -0.4% | 0.6% | 0.3% |
| 1996 | -0.5% | 1.4% | 0.0% | 0.5% | -0.5% | 1.4% | 0.0% | 0.5% |
| 2000 | 2.4% | 1.0% | 1.7% | 1.1% | 2.4% | 1.1% | 1.8% | 1.1% |
| 2004 | -0.1% | 1.0% | -0.3% | 0.7% | -0.1% | 1.1% | -0.3% | 0.7% |
| 2008 | 1.4% | 1.4% | 1.6% | 1.8% | 1.5% | 1.5% | 1.5% | 1.8% |
| 2012 | 1.0% | -0.9% | 0.3% | -0.3% | 1.0% | -0.9% | 0.3% | -0.3% |
| 2016 | 0.8% | 0.8% | -0.2% | -0.5% | 0.9% | 0.8% | -0.2% | -0.5% |
| 2020 | 0.9% | 1.2% | -0.3% | 0.3% | 0.8% | 1.3% | -0.3% | 0.3% |
| 2024 | -0.6% | 0.1% | 0.3% | 0.9% | -0.6% | 0.1% | 0.3% | 0.9% |
| **Total percentage change** | **24.8%** | **12.7%** | **15.0%** | **22.4%** | **33.0%** | **14.5%** | **17.5%** | **28.8%** |

Note. For final performance time, percentage change was calculated as [(previous available Olympic mean − current Olympic mean) / previous available Olympic mean] × 100. For swimming speed, percentage change was calculated as [(current Olympic mean − previous available Olympic mean) / previous available Olympic mean] × 100. Mean values only were used; standard deviations were not included. Thus, positive values indicate either a decrease in final time or an increase in swimming speed. Total percentage change was calculated between the first available Olympic mean and the 2024 Olympic mean using the corresponding formula. F: Female; M: Male; NA: Not available/not applicable because no previous comparable Olympic value was available.

**S9 Table. Percentage changes in reaction times for backstroke, breaststroke, and butterfly final performances across consecutive Olympic Games (2000–2024)**

|  | **Backstroke** | **Backstroke** | **Backstroke** | **Backstroke** | **Breaststroke** | **Breaststroke** |
| --- | --- | --- | --- | --- | --- | --- |
|  | **100m** | **100m** | **200m** | **200m** | **100m** | **100m** |
|  | **F** | **M** | **F** | **M** | **F** | **M** |
| 2000 | NA | NA | NA | NA | NA | NA |
| 2004 | NA | NA | NA | NA | 6.3% | 1.3% |
| 2008 | 1.5% | 3.1% | 4.3% | 1.5% | -1.3% | -1.3% |
| 2012 | 4.5% | -1.6% | -1.5% | 7.5% | 9.2% | 10.5% |
| 2016 | -4.8% | 1.6% | 0.0% | 1.6% | 1.4% | 2.9% |
| 2020 | 10.6% | 8.1% | 10.4% | 3.3% | 1.5% | 4.5% |
| 2024 | 0.0% | -1.8% | 1.7% | -1.7% | -1.5% | -4.8% |
| **Total percentage change** | **11.9%** | **9.4%** | **14.5%** | **11.8%** | **15.0%** | **13.2%** |
|  | **Breaststroke** | **Breaststroke** | **Butterfly** | **Butterfly** | **Butterfly** | **Butterfly** |
|  | **200m** | **200m** | **100m** | **100m** | **200m** | **200m** |
|  | **F** | **M** | **F** | **M** | **F** | **M** |
| 2000 | NA | NA | NA | NA | NA | NA |
| 2004 | 0.0% | 0.0% | 3.8% | 1.3% | -1.2% | 1.3% |
| 2008 | 0.0% | 0.0% | 0.0% | 2.7% | 7.3% | 5.1% |
| 2012 | 8.9% | 10.1% | 6.6% | 4.1% | 2.6% | 9.5% |
| 2016 | -4.2% | 2.8% | 0.0% | 2.9% | 4.1% | -1.5% |
| 2020 | 8.0% | 5.8% | 5.6% | 7.4% | 4.2% | 4.4% |
| 2024 | 0.0% | -3.1% | -1.5% | 0.0% | -5.9% | 0.0% |
| **Total percentage change** | **12.7%** | **15.2%** | **13.9%** | **17.1%** | **11.1%** | **17.7%** |

Note. Percentage change in reaction time was calculated as [(previous available Olympic mean − current Olympic mean) / previous available Olympic mean] × 100, using mean values only; standard deviations were not included. Positive values indicate a decrease in reaction time, whereas negative values indicate an increase. Total percentage change was calculated between the first available Olympic mean and the 2024 Olympic mean using the same formula. F: Female; M: Male; NA: Not available/not applicable because no previous comparable Olympic value was available.
