# Supplementary material for: Performance evolution in Olympic swimming over the last 100 years: A retrospective stroke-based analysis
Source: PLoS One. 2026 Oct 26;21(10):e0361289. doi: 10.1371/journal.pone.0361289 (PMC13704801; doi:10.1371/journal.pone.0361289)
Supplement: Supplementary file 4 [file pone.0361289.s006.docx]

**Response to Reviewers**

Reviewers' comments:

Comments to the Author

Reviewer #1: This manuscript examines the evolution of Olympic swimming performance over a 100-year period (1924-2024), including final times, reaction times, and swimming speed across strokes and distances. The topic is relevant, and the use of a large historical dataset is a clear strength. The manuscript is generally well structured and addresses a question of practical interest for researchers, coaches, and athletes.

However, several methodological and analytical issues limit the strength of the conclusions. Addressing these points would substantially improve the scientific rigor and overall contribution of the study.

The manuscript would benefit from a clearer articulation of its novelty relative to existing literature. Similar longitudinal analyses already exist, and the added value of this study should be more explicitly defined.

Response: We thank the reviewer for this valuable comment. We agree that the novelty and unique contribution of the present study should be more explicitly articulated. Accordingly, the final paragraph of the Introduction has been revised to better distinguish the current study from previous longitudinal investigations.

Specifically, we now emphasize that previous studies have generally focused on specific swimming strokes, limited Olympic periods, selected performance variables, or world-ranking datasets. In contrast, the present study provides a comprehensive evaluation of Olympic swimming performance across a 100-year period (1924-2024), simultaneously examining all four competitive strokes, multiple race distances, final race times, reaction times, and swimming speeds using Olympic finalist data within a single analytical framework.

These revisions have been incorporated into the Introduction to more clearly highlight the originality and added value of the study.

The analysis spans a long time frame (1924-2024), during which major changes occurred in technology, regulations, and training. Although acknowledged in the discussion, these factors are not integrated into the analysis. A more structured approach (e.g., segmentation into historical or technological eras) would improve interpretability.,

Response: We thank the reviewer for this insightful comment. We agree that examining Olympic swimming performance within predefined historical, technological, or regulatory eras may provide additional insights into performance evolution. However, the primary aim of the present study was to provide a comprehensive overview of long-term Olympic swimming performance across the entire 1924-2024 period using a unified analytical framework rather than to formally compare predefined historical eras.

To address the reviewer's concern, we have expanded the Discussion section by explicitly acknowledging that major technological, regulatory, and methodological developments occurred throughout the study period and by highlighting that future studies may benefit from segmenting Olympic swimming history into distinct eras to further examine the contribution of these developments to performance progression.

In addition, we have incorporated this issue into the newly added Limitations section, clarifying that technological, regulatory, and methodological developments were not directly incorporated into the statistical analyses and that the present findings should therefore be interpreted as descriptive of long-term performance evolution rather than as evidence of the isolated effects of specific historical developments.

We believe these revisions improve the interpretation of the findings while remaining consistent with the primary objective of the study.

The use of mean finalist performance may attenuate elite-level differences and be influenced by changes in competitive depth. This choice should be better justified, and complementary analyses (e.g., focusing on winners) would strengthen the manuscript. Given the scope of the dataset, including a table with the evolution of winning performances would provide a clearer picture of peak performance over time.

Response: We thank the reviewer for this valuable comment. We agree that analyses based on Olympic champions may provide additional insight into peak athletic performance. However, the primary objective of the present study was to examine the evolution of elite Olympic swimming performance at the finalist level rather than to focus exclusively on individual winners.

To better justify this methodological choice, we have expanded the Data Processing section. Specifically, we now clarify that Olympic finalists represent the highest level of international swimming performance and provide a broader representation of elite competitive standards than individual winning performances. Furthermore, the use of mean finalist values reduces the influence of exceptional individual achievements and isolated performances, thereby providing a more stable estimate of competitive performance across Olympic cycles.

In addition, we have acknowledged this issue in the newly added Limitations section, noting that the use of mean finalist values rather than winning performances may have attenuated some peak-performance differences that could be observed when focusing exclusively on Olympic champions.

We agree that future investigations focusing specifically on Olympic champions may provide complementary insights into the historical evolution of peak swimming performance.

Percentage changes between Olympic Games are only selectively reported, mainly when described as "dramatic" improvements. A more systematic presentation of these data (e.g., in a table or supplementary material) would improve transparency and allow a better assessment of performance evolution.

Response: We thank the reviewer for this constructive suggestion. We agree that a systematic presentation of percentage changes may provide additional insight into performance evolution. However, given the large scope of the dataset, including multiple swimming strokes, race distances, sexes, and Olympic editions spanning a 100-year period, presenting percentage changes for all consecutive Olympic Games would substantially increase the volume and complexity of the Results section and potentially reduce the overall readability of the manuscript.

For this reason, we elected to report the most notable percentage changes within the text while providing complete performance data for all Olympic Games in the corresponding tables. This approach was intended to balance transparency with clarity of presentation. We have nevertheless revised the manuscript to ensure that terms describing performance changes are used more cautiously and consistently throughout the text.

The statistical analysis relies on comparisons between Olympic editions, which does not fully capture temporal dynamics. The manuscript states that performance evolution is non-linear, but it is unclear whether this was formally tested. The identification of "breakthrough periods appears descriptive. Approaches to model trends or detect change-points should be considered or at least discussed.

Response: We thank the reviewer for this important methodological comment. We agree that formal trend-modeling approaches, such as segmented regression or change-point analyses, may provide additional insight into the temporal dynamics of Olympic swimming performance.

The primary objective of the present study was to compare performance outcomes across Olympic editions and to provide a comprehensive descriptive overview of long-term performance evolution rather than to formally identify statistical change-points. To avoid implying that specific Olympic cycles represent statistically verified transition points, we revised the terminology used throughout the manuscript. Terms such as "breakthrough periods and similar expressions were replaced with more descriptive language (e.g., "Olympic cycles associated with the largest percentage improvements in performance").

In addition, the opening section of the Discussion was revised to emphasize that the identified Olympic cycles coincided with major technological, regulatory, and methodological developments rather than representing formally established change-points. Furthermore, a limitation was added acknowledging that formal trend-modeling and change-point analyses were beyond the scope of the present study and that future research employing such approaches may provide additional insight into the temporal dynamics of Olympic swimming performance.

We believe these revisions clarify the descriptive nature of our findings and address the reviewer's concern regarding the interpretation of performance changes across Olympic history.

Related to this, the presentation of multiple pairwise comparisons in the tables is difficult to interpret and appears inconsistent (e.g., lack of symmetry in reported differences between years-if 100 m freestyle in 2024 differs from 1968, the reverse comparison should also be reflected). Additionally, the relatively small number of significant differences across a century of data is unexpected (e.g., the absence of differences between early and recent Olympic Games (for example between the male 100 free from 1924 and 1996) and should be clarified.

Response: Thank you for this thoughtful comment. The pairwise comparisons reported in the tables were generated directly from the applied post-hoc procedures, and all reported significant differences reflect the outcomes of the statistical analyses without any selective reporting by the authors. We acknowledge that some findings may appear counterintuitive, particularly when Olympic Games separated by long time intervals do not exhibit statistically significant differences. However, statistical significance was determined by the applied analytical procedures and the variability present within the dataset rather than by chronological distance between Olympic editions. Therefore, greater temporal separation between Olympic Games did not necessarily correspond to statistically significant differences. We have carefully rechecked all analyses and confirm that the reported results accurately reflect the outcomes of the statistical procedures used in the study.

Swimming speed is derived directly from time and distance and does not represent an independent variable; its analytical value and importance for this manuscript should be clarified. Reaction time data also appear to be limited to more recent Olympic Games and should be clearly contextualized. For example, stating in the abstract that reaction times improved after 2000 may be misleading if data are only available from that period, although historical differences in starting techniques could/should still be discussed.

Response: Thank you for this valuable comment. We agree that swimming speed is mathematically derived from race time and distance and therefore does not represent an independent performance variable. Nevertheless, swimming speed was included because it provides a standardized metric that facilitates comparisons across events of different distances and swimming strokes. To clarify this rationale, an explanatory statement has been added to the Methods section.

We also agree that reaction-time data were available only for the more recent Olympic Games. To avoid potential misinterpretation, we revised the Abstract and Results sections to clearly indicate that reaction-time analyses were limited to the period for which these data were available. In addition, this issue is now acknowledged in the Limitations section.

The discussion proposes plausible explanations for performance improvements (e.g., technology, training, regulations), but these remain largely inferential. A clearer distinction between association and causality would improve the balance of the conclusions. Additionally, terms such as "dramatic changes" should be better defined (e.g., based on percentage thresholds?).

Response: Thank you for this important comment. We agree that the technological, regulatory, and methodological factors discussed in the manuscript should be interpreted as potential explanations rather than as evidence of direct causality. To address this concern, we carefully revised the Discussion section to distinguish associations from causal interpretations by replacing deterministic language with more cautious expressions such as "coincided with," "were associated with," and "may have contributed." In addition, we explicitly acknowledged in the Limitations section that these factors were not directly incorporated into the statistical analyses and that the findings should therefore be interpreted descriptively.

Regarding the use of terms such as "dramatic changes" or "breakthrough periods," we agree that these expressions may imply a level of statistical verification beyond the scope of the present analyses. Accordingly, we revised the manuscript throughout and replaced such terminology with more neutral descriptions, including "largest percentage improvements," "largest percentage reductions," and "periods associated with substantial performance improvements.

The manuscript would benefit from careful revision to address minor inconsistencies (e.g., repeated sentences, untranslated text, and occasional numerical discrepancies e.g., 100 m male freestyle mean values from 2024 is 47,54 and not 47,67).

Methodological details (such as the use of mean finalist data) should also be more clearly stated, and including key numerical findings in the abstract would improve readability. A paragraph explicitly acknowledging the main limitations of the study is also recommended.

Response: Thank you for these helpful suggestions. We carefully reviewed the manuscript to identify and correct minor inconsistencies, including editorial issues and numerical discrepancies. In particular, the 2024 men's 100-m freestyle mean value identified by the reviewer was rechecked against the original dataset and official Olympic records and has been corrected in the revised manuscript.

To improve methodological transparency, we expanded the Data Processing section by providing a clearer justification for the use of mean finalist performances as representative values for each Olympic Games. We also carefully reviewed the manuscript to improve consistency and clarity throughout the text.

Finally, following the reviewer's recommendation, we added a dedicated Limitations section acknowledging the principal methodological constraints of the study, including the use of mean finalist data, the availability of reaction-time records, and the descriptive nature of the historical analyses. While we did not substantially expand the numerical detail presented in the Abstract in order to maintain readability and comply with journal formatting considerations, we carefully revised the Abstract to improve clarity and consistency with the revised manuscript

Overall, the study has clear potential given the relevance of the topic and the size of the dataset. With improved methodological clarity, more appropriate analytical approaches, and more cautious interpretation of results, it could make a valuable contribution to the literature on swimming performance.

Response: We sincerely thank the reviewer for the thorough evaluation of our manuscript and for the constructive comments provided throughout the review process. We appreciate the recognition of the relevance of the topic and the value of the dataset. The reviewer's suggestions have helped us improve the methodological clarity, interpretation of the findings, and overall presentation of the manuscript. We believe that the revisions undertaken in response to these comments have substantially strengthened the quality and scientific rigor of the study.

Reviewer #2: Reviewer request list for manuscript entitled:

Performance Evolution in Olympic Swimming Over the Last 100 Years: A Retrospective Stroke-Based Analysis

Short title: A Century of Olympic Swimming

Authors:

Ayça Anameriç, Elif Cengizel, Burcu Ertaş Dölek, Hacı Ahmet Pekel

The manuscript entitled: Performance Evolution in Olympic Swimming Over the Last 100 Years: A Retrospective Stroke-Based Analysis, can be accepted as interesting and important for the field of Sports Science in terms of its topic, but it is necessary to make the following corrections to the text and changes to its content:

1. Introduction:

a. Line 56-110:

Comment: The introduction is written quite correctly, where the scientific basis of the topic is elaborated in an acceptable manner. Most of the published scientific literature with a similar research topic is covered, based on which the goal of the current manuscript is defined.

Response: We thank the reviewer for the positive evaluation of the Introduction section. We appreciate the recognition that the scientific background, relevant literature, and study rationale were presented appropriately.

2. Methods:

a. Line 121-122: Ethical approval for the study was obtained from the XXX University Ethics Commission (Research Code: 2022-1074)

Comment: The authors did not define the Ethical Permission data's at fully, and instead XXX remained in the text of the paper, should be corrected it.

Response: We thank the reviewer for identifying this oversight. The placeholder text ("XXX University") has been replaced with the full name of the institution, and the ethical approval information is now reported correctly in the revised manuscript

b. Line 2222 Data Processing: For each Olympic Games between 1924 and 2024, mean and standard deviation values were calculated for all finalists' performance variables. Percentage changes in swimming performance between consecutive Olympic Games were calculated by dividing the difference between two successive Games by the performance value of the earlier Games and multiplying the result by 100. Total percentage reduction was calculated 133 by subtracting the mean final performance of the 2024 Olympic Games from the mean final performance of the first Olympic Games in which the stroke was contested, dividing this difference 100.

by the initial Olympic mean, and multiplying the result by Comment: Although the authors explained in text how and from where they calculated the percentage differences in results, the procedure for calculating the percentages is still very unclear. Therefore, it is necessary to give a practical example of their calculation, in order to better understand the main procedure for defining the differences in results between the Olympic Games that served the authors as the main numerical record and evidence of the progress of swimming results.

Response: We thank the reviewer for this suggestion. The percentage-change calculations reported in the manuscript were based on standard percentage-difference formulas and were described in the Data Processing section. To improve clarity, we carefully revised this section and expanded the methodological description of the data-processing procedures. However, we elected not to include a specific numerical example in order to maintain the conciseness of the Methods section and because the same calculation procedure was applied consistently across all swimming events, distances, and Olympic Games analyzed in the study. We hope that the revised description provides sufficient clarity regarding the calculation of percentage changes and total percentage reductions.

3. Results:

a. Line 556-Table 3, and Line 569 Table 4, Line 609-Table 6, Line 625 - Table 7, Line 641 - Table

8.

Comment: Although speed is a linear analogy to swimming time, it is important to represent it as precisely as possible, which the notation used by the authors (to the second decimal place) does not represent. Therefore, it is necessary to write all speeds in the form of the third decimal place (both the average value and the standard deviation), in Tables, but also in all text of manuscript.

Comment: It is necessary to technically unify all tables so that all time results achieved, and reaction times achieved, in all disciplines in all analyzed techniques must be written to the second decimal place (both the average value and the standard deviation).

Response: We thank the reviewer for this helpful suggestion. To improve numerical precision and consistency throughout the manuscript, all swimming-speed values (means and standard deviations) have been revised and are now reported to three decimal places in the relevant tables and corresponding text. In addition, all race-time and reaction-time values (means and standard deviations) have been standardized and are now consistently presented to two decimal places throughout the manuscript. We believe that these revisions improve both the precision and the overall consistency of data presentation.

4. Disscussion:

a. Line 264 till 267: The findings of the present study revealed that these performance breakthroughs were particularly pronounced during specific Olympic cycles, such as 1956, 1972, and 2008, corresponding with major technological and methodological advancements in the sport.

Comment: Unfortunately, the authors did not provide statistically specific evidence that these years are the most representative for the progress of Olympic swimming results. Evidence of this claim should be included in the form of specific statistical analyses and parameters, either in the results section or in parentheses after this sentence with the given claim.

Response: We thank the reviewer for this important observation. We agree that the Olympic Games highlighted in the original version of the manuscript (e.g., 1956, 1972, and 2008) were identified descriptively based on the largest observed percentage improvements rather than through formal change-point or trend-detection analyses. Accordingly, we revised the manuscript throughout to avoid terminology implying statistically verified "breakthrough" periods. In the revised Discussion section, we explicitly clarify that these Olympic Games should be interpreted as periods associated with the largest percentage performance improvements observed in the dataset rather than as statistically confirmed transition points. We also added a statement acknowledging that formal longitudinal modeling approaches, such as segmented regression or change-point analyses, may provide additional insight in future investigations.

b. Line 267271: For instance, the introduction of refined training systems, the evolution of hydrodynamic swimsuit designs, improvements in pool construction (lane lines, depth, water circulation), and stricter starting regulations likely contributed to substantial time reductions observed in freestyle (1956, 1972, 2008), backstroke (1928, 1956, 1972, 2008), breaststroke (1972, 1976, 2008), and butterfly events (1964, 1972, 1984).

Comment: Like the previous comment, the authors did not provide statistically specific evidence that these years are the most representative for the progress of Olympic swimming results. considering different swimming strokes. Evidence of this claim should be included in the form of specific statistical analyses and parameters, either in the results section or in parentheses after this sentence with the given claim.

Response: We thank the reviewer for this observation. We agree that the wording used in the original manuscript could be interpreted as implying that the identified Olympic Games represented statistically verified periods of performance progression across different swimming strokes. As noted in our response to the previous comment, these Olympic Games were identified descriptively based on the largest observed percentage improvements rather than through formal change-point analyses or other statistical procedures designed to identify transition periods.

Accordingly, the cited sentence has been revised in the Discussion section. References to specific Olympic Games as "breakthrough" periods have been removed, and the discussion has been reformulated to emphasize that these observations represent descriptive patterns within the confirmed transition points. than rather dataset statistically

c. Although the authors presented the results of reaction times in Table 5 and Table 9, in the Discussion section they did not comment or discuss the obtained results, so it is necessary to insert the same in the discussion with all the scientific explanations of the established trend of change of the given phenomenon.

Response: We thank the reviewer for this valuable comment. We agree that the reaction-time findings were insufficiently discussed in the original version of the manuscript. Accordingly, we added a dedicated subsection ("Reaction Time") to the Discussion section. In this new section, we discuss the observed reaction-time trends across the Olympic Games for which reaction-time data were available and provide potential explanations related to developments in start techniques, competition technology, and start-specific training practices. We believe that this addition improves the interpretation and contextualization of the reaction-time findings presented in Tables 5 and 9.

5. Conclusion: The Conclusion section is written in acceptable form and content. If all the required details are satisfactorily corrected in the revision of the work, the conclusion can remain as it is.

Response: We thank the reviewer for the positive evaluation of the Conclusion section. We appreciate the reviewer's assessment and have carefully addressed all comments and suggestions provided throughout the review process. We believe that the revisions undertaken have strengthened the manuscript and improved its overall clarity and scientific rigor

6. Acknowledgments:

a. Line 385: A part of this research was presented as an oral presentation at the XXXX Congress.

b. Comment: XXXX Congres It is necessary for the authors to accurately state all the data about the presented paper and the congress.

Response: We thank the reviewer for identifying this omission. The placeholder text ("XXXX Congress") has been removed and replaced with the complete conference information. The Acknowledgments section has been revised to indicate that part of this research was presented as an oral presentation at the 15th National Sports Science Student Congress, Ankara, Turkey, 8-9 May 2023.

7. References: References are adequately selected and currently cover the topic of the manuscript. However, references numbered: 5, 29, 32 and 34 are not adequately cited according to technical standards, so they need to be adequately technically cited.

Response: We thank the reviewer for identifying these reference-formatting issues. References 5, 29, 32, and 34 have been carefully checked and revised according to the technical citation requirements. The book chapter, master's thesis, journal article, and conference proceeding have now been cited with the appropriate bibliographic details, including editors, publication type, publisher/institution, journal information, page range, DOI, and/or availability information where applicable.

General comments:

General

1. The main weakness in the manuscript is the lack of any formal statistical evidence for the authors' claim that the years of critical progress for swimming in individual techniques are exactly those that the authors claim. Namely, the authors only presented in the Tables in the form of letter markings the formally established statistical difference between the results of the final races at the analyzed Olympics, where no other regular statistical evidence is presented such as (F value, effect size, power of analysis, etc...). It is simply not clear on what basis the authors concluded about the statistical significance of the selected years, as representatively critical in terms of the development of Olympic swimming results. As such analyses implemented in the current manuscript are very important for the science of swimming, as well as the development of the methodology of scientific research work, it is necessary that the authors fully present the statistical evidence of the given claims, as a criterion evidence for defining the scientific conclusion of the study. As long as the manuscript remains in its current form, and if it is not improved in terms of the comments given, I believe that it is not suitable for acceptance in terms of valid fundamental scientific research.

Response: We sincerely thank the reviewer for this detailed and thoughtful comment. We agree that the wording used in the original version of the manuscript may have implied that specific Olympic Games (e.g., 1956, 1972, and 2008) represented statistically verified transition points in the historical progression of Olympic swimming performance. However, the primary purpose of the present study was to provide a descriptive historical analysis of long-term Olympic swimming performance rather than to formally identify change-points through advanced longitudinal modeling techniques.

In response to the reviewer's concern, we carefully revised the manuscript to avoid language suggesting statistically confirmed "breakthrough periods. References to specific Olympic Games as critical or breakthrough years have been replaced with more descriptive terminology such as "Olympic Games associated with the largest percentage performance improvements." In addition, we added explicit statements in the Discussion and Limitations sections clarifying that the present analyses were not designed to formally model temporal trends or identify statistical change-points and that the highlighted Olympic Games should therefore be interpreted descriptively rather than as statistically verified transition points.

We believe that these revisions provide a more accurate representation of the scope of the analyses performed and align the interpretation of the findings with the statistical methods employed in the study.

We sincerely thank the reviewer for the careful and constructive evaluation of our manuscript. We greatly appreciate the detailed comments and methodological observations provided throughout the review process. The reviewer's suggestions have helped us improve the clarity of the data-processing procedures, the interpretation of the findings, the presentation of the results, and the overall scientific rigor of the manuscript. We believe that the revisions undertaken in response to these comments have substantially strengthened the quality, transparency, and readability of the study. We are grateful for the time and expertise devoted to reviewing our work
