# Supplementary material for: Performance evolution in Olympic swimming over the last 100 years: A retrospective stroke-based analysis
Source: PLoS One. 2026 Oct 26;21(10):e0361289. doi: 10.1371/journal.pone.0361289 (PMC13704801; doi:10.1371/journal.pone.0361289)
Supplement: Supplementary file 5 [file pone.0361289.s003.docx]

Reviewer request list for manuscript entitled:

Performance Evolution in Olympic Swimming Over the Last 100 Years: A RetrospectiveStroke-Based Analysis

Short title: A Century of Olympic Swimming

Authors:

Ayça Anameriç, Elif Cengizel, Burcu Ertaş Dölek, Hacı Ahmet Pekel

The manuscript entitled: Performance Evolution in Olympic Swimming Over the Last 100 Years: A RetrospectiveStroke-Based Analysis, can be accepted as interesting and important for the field of Sports Science in terms of its topic, but it is necessary to make the following corrections to the text and changes to its content:

1. Introduction:
   a. Line 56 – 110:

Comment: The introduction is written quite correctly, where the scientific basis of the topic is elaborated in an acceptable manner. Most of the published scientific literature with a similar research topic is covered, based on which the goal of the current manuscript is defined.

2. Methods:
   a. Line 121-122: Ethical approval for the study was obtained from the XXX University Ethics Commission (Research Code: 2022-1074)

Comment: The authors did not define the Ethical Permission data’s at fully, and	instead XXX remained in the text of the paper, should be corrected it.

b. Line 2222 - Data Processing: For each Olympic Games between 1924 and 2024, mean and standard deviation values were calculated for all finalists’ performance variables. Percentage changes in swimming performance between consecutive Olympic Games were calculated by dividing the difference between two successive Games by the performance value of the earlier Games and multiplying the result by 100. Total percentage reduction was calculated 133 by subtracting the mean final performance of the 2024 Olympic Games from the mean final performance of the first Olympic Games in which the stroke was contested, dividing this difference by the initial Olympic mean, and multiplying the result by 100.

Comment: Although the authors explained in text how and from where they	calculated the percentage differences in results, the procedure for calculating the	percentages is still very unclear. Therefore, it is necessary to give a practical example	of their calculation, in order to better understand the main procedure for defining	the differences in results between the Olympic Games that served the authors as the	main numerical record and evidence of the progress of swimming results.

3. Results:
   a. Line 556 - Table 3, and Line 569 - Table 4, Line 609 - Table 6, Line 625 - Table 7, Line 641 - Table 8.

Comment: Although speed is a linear analogy to swimming time, it is important to represent it as precisely as possible, which the notation used by the authors (to the second decimal place) does not represent. Therefore, it is necessary to write all speeds in the form of the third decimal place (both the average value and the standard deviation), in Tables, but also in all text of manuscript.

Comment: It is necessary to technically unify all tables so that all time results achieved, and reaction times achieved, in all disciplines in all analyzed techniques must be written to the second decimal place (both the average value and the standard deviation).

4. Disscussion:
   a. Line 264 till 267: The findings of the present study revealed that these performance breakthroughs were particularly pronounced during specific Olympic cycles, such as 1956, 1972, and 2008, corresponding with major technological and methodological advancements in the sport.

Comment: Unfortunately, the authors did not provide statistically specific evidence that these years are the most representative for the progress of Olympic swimming results. Evidence of this claim should be included in the form of specific statistical analyses and parameters, either in the results section or in parentheses after this sentence with the given claim.

b. Line 267 – 271: For instance, the introduction of refined training systems, the evolution of hydrodynamic swimsuit designs, improvements in pool construction (lane lines, depth, water circulation), and stricter starting regulations likely contributed to substantial time reductions observed in freestyle (1956, 1972, 2008), backstroke (1928, 1956, 1972, 2008), breaststroke (1972, 1976, 2008), and butterfly events (1964, 1972, 1984).

Comment: Like the previous comment, the authors did not provide statistically	specific evidence that these years are the most representative for the progress of	Olympic swimming results considering different swimming strokes. Evidence of this	claim should be included in the form of specific statistical analyses and parameters,	either in the results section or in parentheses after this sentence with the given	claim.

c. Although the authors presented the results of reaction times in Table 5 and Table 9, in the Discussion section they did not comment or discuss the obtained results, so it is necessary to insert the same in the discussion with all the scientific explanations of the established trend of change of the given phenomenon..

5. Conclusion: The Conclusion section is written in acceptable form and content. If all the required details are satisfactorily corrected in the revision of the work, the conclusion can remain as it is.
6. Acknowledgments:
   a. Line 385: A part of this research was presented as an oral presentation at the XXXX Congress.
   b. Comment: XXXX Congres - It is necessary for the authors to accurately state all the data about the presented paper and the congress.
7. References: References are adequately selected and currently cover the topic of the manuscript. However, references numbered: 5, 29, 32 and 34 are not adequately cited according to technical standards, so they need to be adequately technically cited.General comments:

General

1. The main weakness in the manuscript is the lack of any formal statistical evidence for the authors' claim that the years of critical progress for swimming in individual techniques are exactly those that the authors claim. Namely, the authors only presented in the Tables in the form of letter markings the formally established statistical difference between the results of the final races at the analyzed Olympics, where no other regular statistical evidence is presented such as (F value, effect size, power of analysis, etc...). It is simply not clear on what basis the authors concluded about the statistical significance of the selected years, as representatively critical in terms of the development of Olympic swimming results. As such analyses implemented in the current manuscript are very important for the science of swimming, as well as the development of the methodology of scientific research work, it is necessary that the authors fully present the statistical evidence of the given claims, as a criterion evidence for defining the scientific conclusion of the study. As long as the manuscript remains in its current form, and if it is not improved in terms of the comments given, I believe that it is not suitable for acceptance in terms of valid fundamental scientific research.
