# Supplementary material for: Performance evolution in Olympic swimming over the last 100 years: A retrospective stroke-based analysis
Source: PLoS One. 2026 Oct 26;21(10):e0361289. doi: 10.1371/journal.pone.0361289 (PMC13704801; doi:10.1371/journal.pone.0361289)
Supplement: Supplementary file 6 [file pone.0361289.s005.docx]

- Table 3. Line 625,
  - 100m Female, 1964, please corect averaged velocity number - 1.627-3±0.033
  - Please, do correct for the font numbers to 2024 yrs.
- Table 4. Line 637,
  - Please, do correct for the font numbers to 2024 yrs.
- Table 5. Line 651,
  - Please, do correct for the font numbers to the discipline 400, 800 and 1500m.
- Table 9. Line 719,
  - Please, do correct for the font numbers to 2024 yrs.
