# Supplementary material for: Performance evolution in Olympic swimming over the last 100 years: A retrospective stroke-based analysis
Source: PLoS One. 2026 Oct 26;21(10):e0361289. doi: 10.1371/journal.pone.0361289 (PMC13704801; doi:10.1371/journal.pone.0361289)
Supplement: Supplementary file 7 [file pone.0361289.s007.docx]

Dear Editor and Reviewers,

We sincerely thank the Editor and the Reviewers for their careful evaluation of our manuscript and for their constructive comments and suggestions. We have carefully addressed all comments raised during this revision round and revised the manuscript accordingly. Our point-by-point responses are provided below, together with details of the corresponding changes made to the manuscript.

Reviewer #1: Thank you for the detailed responses and the revisions made to the manuscript. The paper has clearly improved, especially regarding the clarification of its novelty, the methodological explanations, the more cautious interpretation of the results, and the addition of a limitations section.
I only have a few minor comments before publication:

1. The explanation about the pairwise comparisons could be expanded slightly. Some non-significant differences between Olympic Games separated by several decades still seem unexpected. A much better explanation of how the variability in the data and the multiple-comparison procedure may have influenced these results would help readers understand these findings.

Response: We thank the reviewer for this helpful comment. We agree that the interpretation of the pairwise comparisons required further clarification. Accordingly, we have expanded the Discussion to clarify that the absence of statistically significant differences between some Olympic Games separated by several decades does not necessarily indicate an absence of long-term performance improvement. We now explain that the statistical significance of pairwise comparisons depends not only on the magnitude of the differences between Olympic editions but also on within-edition variability and the multiple-comparison procedure applied. Therefore, chronological distance between Olympic Games does not necessarily correspond directly to statistical significance. In addition, the Data Analysis section has been revised to specify that post hoc pairwise comparisons were performed using Holm–Sidak, Tukey, or Dunn’s procedures. We believe these additions provide clearer context for interpreting the pairwise comparison results.

2. The presentation of the percentage changes between consecutive Olympic Games should also be improved. Since the tables already include the mean performance values for each Olympic edition, I suggest adding one extra column showing the percentage change compared with the previous Olympic Games (or providing these data as supplementary material if space is an issue). This would make the evolution of performance much easier to follow without requiring any additional analyses.

Overall, I believe the authors have addressed the main concerns raised during the first review, and I consider the manuscript suitable for publication after these minor revisions.

Response: We thank the reviewer for this helpful suggestion. In response, we have added Supplementary Tables S1–S9 presenting the percentage changes between consecutive available Olympic Games for final performance times, swimming speeds, and reaction times, as applicable, across all events and both sexes. This supplementary material allows the temporal changes in performance to be followed more clearly without further expanding the main tables. We have also added a corresponding statement in the Results section directing readers to these supplementary tables: “Percentage changes between consecutive available Olympic Games for final performance times, swimming speeds, and reaction times are presented in S1–S9 Tables.

Reviewer #2: A very significant work for the science and practice of top-level sports and swimming. Such research represents the foundation for summarizing knowledge about the development of a sport.

Response: We sincerely thank the reviewer for this positive and encouraging assessment of our work and for recognizing the potential contribution of our study to the understanding of the long-term development of competitive swimming.

Reviewer #2 – Additional Technical Corrections

Table 3. Line 625,

- 100m Female, 1964, please corect averaged velocity number - 1.627-3±0.033

Response: Thank you for identifying this typographical error. The mean swimming speed value for the women’s 100-m freestyle event at the 1964 Olympic Games in Table 3 has been corrected.

- Please, do correct for the font numbers to 2024 yrs.

Response: Thank you. The font formatting of the values through the 2024 Olympic Games in Table 3 has been corrected and standardized.

Table 4. Line 637,

- Please, do correct for the font numbers to 2024 yrs.

Response: Thank you. The font formatting of the values through the 2024 Olympic Games in Table 4 has been corrected and standardized.

Table 5. Line 651,

- Please, do correct for the font numbers to the discipline 400, 800 and 1500m.

Response: Thank you. The font formatting of the values for the 400-, 800-, and 1500-m events in Table 5 has been corrected and standardized.

Table 9. Line 719,

- Please, do correct for the font numbers to 2024 yrs.

Response: Thank you. The font formatting of the values through the 2024 Olympic Games in Table 9 has been corrected and standardized.
