# Supplementary material for: Performance evolution in Olympic swimming over the last 100 years: A retrospective stroke-based analysis
Source: PLoS One. 2026 Oct 26;21(10):e0361289. doi: 10.1371/journal.pone.0361289 (PMC13704801; doi:10.1371/journal.pone.0361289)
Supplement: Peer review history

# Peer review: Decision Letter 0

**DOI:** 10.1371/journal.pone.0361289.r001

**Authors:** Dalton Pessôa Filho

27 Apr 2026

Dear Dr. Cengizel,

Thank you for submitting your manuscript to PLOS ONE. After careful consideration, we feel that it has merit but does not fully meet PLOS ONE’s publication criteria as it currently stands. Therefore, we invite you to submit a revised version of the manuscript that addresses the points raised during the review process.

Please submit your revised manuscript by Jun 11 2026 11:59PM. If you will need more time than this to complete your revisions, please reply to this message or contact the journal office at plosone@plos.org. When you're ready to submit your revision, log on to https://www.editorialmanager.com/pone/ and select the 'Submissions Needing Revision' folder to locate your manuscript file.

- A letter that responds to each point raised by the academic editor and reviewer(s). You should upload this letter as a separate file labeled 'Response to Reviewers'.
- A marked-up copy of your manuscript that highlights changes made to the original version. You should upload this as a separate file labeled 'Revised Manuscript with Track Changes'.
- An unmarked version of your revised paper without tracked changes. You should upload this as a separate file labeled 'Manuscript'.

If you would like to make changes to your financial disclosure, please include your updated statement in your cover letter. Guidelines for resubmitting your figure files are available below the reviewer comments at the end of this letter.

If applicable, we recommend that you deposit your laboratory protocols in protocols.io to enhance the reproducibility of your results. Protocols.io assigns your protocol its own identifier (DOI) so that it can be cited independently in the future. For instructions see: https://journals.plos.org/plosone/s/submission-guidelines#loc-laboratory-protocols. Additionally, PLOS ONE offers an option for publishing peer-reviewed Lab Protocol articles, which describe protocols hosted on protocols.io. Read more information on sharing protocols at https://plos.org/protocols?utm_medium=editorial-email&utm_source=authorletters&utm_campaign=protocols.

As the corresponding author, your ORCID iD is verified in the submission system and will appear in the published article. PLOS supports the use of ORCID, and we encourage all coauthors to register for an ORCID iD and use it as well. Please encourage your coauthors to verify their ORCID iD within the submission system before final acceptance, as unverified ORCID iDs will not appear in the published article. *Only*  the individual author can complete the verification step; PLOS staff *cannot*  verify ORCID iDs on behalf of authors.

We look forward to receiving your revised manuscript.

Kind regards,

Dalton Müller Pessôa Filho, Ph.D.

Academic Editor

PLOS One

Journal Requirements:

When submitting your revision, we need you to address these additional requirements.

1. Please ensure that your manuscript meets PLOS ONE's style requirements, including those for file naming. The PLOS ONE style templates can be found at

https://journals.plos.org/plosone/s/file?id=wjVg/PLOSOne_formatting_sample_main_body.pdf and

https://journals.plos.org/plosone/s/file?id=ba62/PLOSOne_formatting_sample_title_authors_affiliations.pdf

2. Thank you for uploading your study's underlying data set. Unfortunately, the repository you have noted in your Data Availability statement does not qualify as an acceptable data repository according to PLOS's standards.

At this time, please upload the minimal data set necessary to replicate your study's findings to a stable, public repository (such as figshare or Dryad) and provide us with the relevant URLs, DOIs, or accession numbers that may be used to access these data. For a list of recommended repositories and additional information on PLOS standards for data deposition, please see https://journals.plos.org/plosone/s/recommended-repositories.

3. Your ethics statement should only appear in the Methods section of your manuscript. If your ethics statement is written in any section besides the Methods, please delete it from any other section.

4. If the reviewer comments include a recommendation to cite specific previously published works, please review and evaluate these publications to determine whether they are relevant and should be cited. There is no requirement to cite these works unless the editor has indicated otherwise.

Additional Editor Comments:

Dear authors, after this first round of revision, the reviewers have some comments/suggestions which I recommend to be deemed by the author(s) before the manuscript final recommendation for publication

[Note: HTML markup is below. Please do not edit.]

Reviewers' comments:

Reviewer's Responses to Questions

**Comments to the Author**

1. Is the manuscript technically sound, and do the data support the conclusions?

Reviewer #1: Partly

Reviewer #2: Partly

**********

2. Has the statistical analysis been performed appropriately and rigorously?

Reviewer #1: Yes

Reviewer #2: No

**********

3. Have the authors made all data underlying the findings in their manuscript fully available?

The PLOS Data policy

Reviewer #1: Yes

Reviewer #2: Yes

**********

4. Is the manuscript presented in an intelligible fashion and written in standard English?

Reviewer #1: Yes

Reviewer #2: Yes

**********

Reviewer #1: This manuscript examines the evolution of Olympic swimming performance over a 100-year period (1924–2024), including final times, reaction times, and swimming speed across strokes and distances. The topic is relevant, and the use of a large historical dataset is a clear strength. The manuscript is generally well structured and addresses a question of practical interest for researchers, coaches, and athletes.

However, several methodological and analytical issues limit the strength of the conclusions. Addressing these points would substantially improve the scientific rigor and overall contribution of the study.

The manuscript would benefit from a clearer articulation of its novelty relative to existing literature. Similar longitudinal analyses already exist, and the added value of this study should be more explicitly defined.

The analysis spans a long time frame (1924–2024), during which major changes occurred in technology, regulations, and training. Although acknowledged in the discussion, these factors are not integrated into the analysis. A more structured approach (e.g., segmentation into historical or technological eras) would improve interpretability.

The use of mean finalist performance may attenuate elite-level differences and be influenced by changes in competitive depth. This choice should be better justified, and complementary analyses (e.g., focusing on winners) would strengthen the manuscript. Given the scope of the dataset, including a table with the evolution of winning performances would provide a clearer picture of peak performance over time.

Percentage changes between Olympic Games are only selectively reported, mainly when described as “dramatic” improvements. A more systematic presentation of these data (e.g., in a table or supplementary material) would improve transparency and allow a better assessment of performance evolution.

The statistical analysis relies on comparisons between Olympic editions, which does not fully capture temporal dynamics. The manuscript states that performance evolution is non-linear, but it is unclear whether this was formally tested. The identification of “breakthrough” periods appears descriptive. Approaches to model trends or detect change-points should be considered or at least discussed.

Related to this, the presentation of multiple pairwise comparisons in the tables is difficult to interpret and appears inconsistent (e.g., lack of symmetry in reported differences between years—if 100 m freestyle in 2024 differs from 1968, the reverse comparison should also be reflected). Additionally, the relatively small number of significant differences across a century of data is unexpected (e.g., the absence of differences between early and recent Olympic Games (for example between the male 100 free from 1924 and 1996) and should be clarified.

Swimming speed is derived directly from time and distance and does not represent an independent variable; its analytical value and importance for this manuscript should be clarified. Reaction time data also appear to be limited to more recent Olympic Games and should be clearly contextualized. For example, stating in the abstract that reaction times improved after 2000 may be misleading if data are only available from that period, although historical differences in starting techniques could/should still be discussed.

The discussion proposes plausible explanations for performance improvements (e.g., technology, training, regulations), but these remain largely inferential. A clearer distinction between association and causality would improve the balance of the conclusions. Additionally, terms such as “dramatic changes” should be better defined (e.g., based on percentage thresholds?).

The manuscript would benefit from careful revision to address minor inconsistencies (e.g., repeated sentences, untranslated text, and occasional numerical discrepancies - e.g., 100 m male freestyle mean values from 2024 is 47,54 and not 47,67).

Methodological details (such as the use of mean finalist data) should also be more clearly stated, and including key numerical findings in the abstract would improve readability.

A paragraph explicitly acknowledging the main limitations of the study is also recommended.

Overall, the study has clear potential given the relevance of the topic and the size of the dataset. With improved methodological clarity, more appropriate analytical approaches, and more cautious interpretation of results, it could make a valuable contribution to the literature on swimming performance.

Reviewer #2: Reviewer request list for manuscript entitled:

Performance Evolution in Olympic Swimming Over the Last 100 Years: A RetrospectiveStroke-Based Analysis

Short title: A Century of Olympic Swimming

Authors:

Ayça Anameriç, Elif Cengizel, Burcu Ertaş Dölek, Hacı Ahmet Pekel

The manuscript entitled: Performance Evolution in Olympic Swimming Over the Last 100 Years: A RetrospectiveStroke-Based Analysis, can be accepted as interesting and important for the field of Sports Science in terms of its topic, but it is necessary to make the following corrections to the text and changes to its content:

1. Introduction:

a. Line 56 – 110:

Comment: The introduction is written quite correctly, where the scientific basis of the topic is elaborated in an acceptable manner. Most of the published scientific literature with a similar research topic is covered, based on which the goal of the current manuscript is defined.

2. Methods:

a. Line 121-122: Ethical approval for the study was obtained from the XXX University Ethics Commission (Research Code: 2022-1074)

Comment: The authors did not define the Ethical Permission data’s at fully, and instead XXX remained in the text of the paper, should be corrected it.

b. Line 2222 - Data Processing: For each Olympic Games between 1924 and 2024, mean and standard deviation values were calculated for all finalists’ performance variables. Percentage changes in swimming performance between consecutive Olympic Games were calculated by dividing the difference between two successive Games by the performance value of the earlier Games and multiplying the result by 100. Total percentage reduction was calculated 133 by subtracting the mean final performance of the 2024 Olympic Games from the mean final performance of the first Olympic Games in which the stroke was contested, dividing this difference by the initial Olympic mean, and multiplying the result by 100.

Comment: Although the authors explained in text how and from where they calculated the percentage differences in results, the procedure for calculating the percentages is still very unclear. Therefore, it is necessary to give a practical example of their calculation, in order to better understand the main procedure for defining the differences in results between the Olympic Games that served the authors as the main numerical record and evidence of the progress of swimming results.

3. Results:

a. Line 556 - Table 3, and Line 569 - Table 4, Line 609 - Table 6, Line 625 - Table 7, Line 641 - Table 8.

Comment: Although speed is a linear analogy to swimming time, it is important to represent it as precisely as possible, which the notation used by the authors (to the second decimal place) does not represent. Therefore, it is necessary to write all speeds in the form of the third decimal place (both the average value and the standard deviation), in Tables, but also in all text of manuscript.

Comment: It is necessary to technically unify all tables so that all time results achieved, and reaction times achieved, in all disciplines in all analyzed techniques must be written to the second decimal place (both the average value and the standard deviation).

4. Disscussion:

a. Line 264 till 267: The findings of the present study revealed that these performance breakthroughs were particularly pronounced during specific Olympic cycles, such as 1956, 1972, and 2008, corresponding with major technological and methodological advancements in the sport.

Comment: Unfortunately, the authors did not provide statistically specific evidence that these years are the most representative for the progress of Olympic swimming results. Evidence of this claim should be included in the form of specific statistical analyses and parameters, either in the results section or in parentheses after this sentence with the given claim.

b. Line 267 – 271: For instance, the introduction of refined training systems, the evolution of hydrodynamic swimsuit designs, improvements in pool construction (lane lines, depth, water circulation), and stricter starting regulations likely contributed to substantial time reductions observed in freestyle (1956, 1972, 2008), backstroke (1928, 1956, 1972, 2008), breaststroke (1972, 1976, 2008), and butterfly events (1964, 1972, 1984).

Comment: Like the previous comment, the authors did not provide statistically specific evidence that these years are the most representative for the progress of Olympic swimming results considering different swimming strokes. Evidence of this claim should be included in the form of specific statistical analyses and parameters, either in the results section or in parentheses after this sentence with the given claim.

c. Although the authors presented the results of reaction times in Table 5 and Table 9, in the Discussion section they did not comment or discuss the obtained results, so it is necessary to insert the same in the discussion with all the scientific explanations of the established trend of change of the given phenomenon..

5. Conclusion: The Conclusion section is written in acceptable form and content. If all the required details are satisfactorily corrected in the revision of the work, the conclusion can remain as it is.

6. Acknowledgments:

a. Line 385: A part of this research was presented as an oral presentation at the XXXX Congress.

b. Comment: XXXX Congres - It is necessary for the authors to accurately state all the data about the presented paper and the congress.

7. References: References are adequately selected and currently cover the topic of the manuscript. However, references numbered: 5, 29, 32 and 34 are not adequately cited according to technical standards, so they need to be adequately technically cited.General comments:

General

1. The main weakness in the manuscript is the lack of any formal statistical evidence for the authors' claim that the years of critical progress for swimming in individual techniques are exactly those that the authors claim. Namely, the authors only presented in the Tables in the form of letter markings the formally established statistical difference between the results of the final races at the analyzed Olympics, where no other regular statistical evidence is presented such as (F value, effect size, power of analysis, etc...). It is simply not clear on what basis the authors concluded about the statistical significance of the selected years, as representatively critical in terms of the development of Olympic swimming results. As such analyses implemented in the current manuscript are very important for the science of swimming, as well as the development of the methodology of scientific research work, it is necessary that the authors fully present the statistical evidence of the given claims, as a criterion evidence for defining the scientific conclusion of the study. As long as the manuscript remains in its current form, and if it is not improved in terms of the comments given, I believe that it is not suitable for acceptance in terms of valid fundamental scientific research.

**********

what does this mean?). If published, this will include your full peer review and any attached files.

If you choose “no”, your identity will remain anonymous but your review may still be made public.

**Do you want your identity to be public for this peer review?**  For information about this choice, including consent withdrawal, please see our Privacy Policy

Reviewer #1: No

Reviewer #2: No

**********

[NOTE: If reviewer comments were submitted as an attachment file, they will be attached to this email and accessible via the submission site. Please log into your account, locate the manuscript record, and check for the action link "View Attachments". If this link does not appear, there are no attachment files.]

To ensure your figures meet our technical requirements, please review our figure guidelines: https://journals.plos.org/plosone/s/figures

You may also use PLOS’s free figure tool, NAAS, to help you prepare publication quality figures: https://journals.plos.org/plosone/s/figures#loc-tools-for-figure-preparation.

NAAS will assess whether your figures meet our technical requirements by comparing each figure against our figure specifications.

Attachment Submitted filename: Reviewer request list_RW_MD_20042026_Plos ONE.docx

# Author comment: Author response to Decision Letter 1

**DOI:** 10.1371/journal.pone.0361289.r002

1 Jul 2026

We thank the Academic Editor and the reviewers for their constructive comments and suggestions. We have carefully revised the manuscript in accordance with all recommendations and have provided a detailed point-by-point response in the uploaded “Response to Reviewers” document. Major revisions include improvements to the methodological descriptions, clarification of the interpretation of performance trends, revision of the Discussion and Conclusion sections, addition of a Limitations section, discussion of reaction-time findings, correction of identified inconsistencies, updating of references, and provision of a publicly accessible minimal dataset via Figshare. We believe that these revisions have substantially strengthened the manuscript and improved its clarity, transparency, and scientific rigor.

Attachment Submitted filename: Response to Reviewers.docx

# Peer review: Decision Letter 1

**DOI:** 10.1371/journal.pone.0361289.r003

**Authors:** Dalton Pessôa Filho

31 Aug 2026

Dear Dr. Cengizel,

Thank you for submitting your manuscript to PLOS One. After careful consideration, we feel that it has merit but does not fully meet PLOS One’s publication criteria as it currently stands. Therefore, we invite you to submit a revised version of the manuscript that addresses the points raised during the review process.

Please submit your revised manuscript by Oct 15 2026 11:59PM. If you will need more time than this to complete your revisions, please reply to this message or contact the journal office at plosone@plos.org. When you're ready to submit your revision, log on to https://www.editorialmanager.com/pone/ and select the 'Submissions Needing Revision' folder to locate your manuscript file.

- A letter that responds to each point raised by the academic editor and reviewer(s). You should upload this letter as a separate file labeled 'Response to Reviewers'.
- A marked-up copy of your manuscript that highlights changes made to the original version. You should upload this as a separate file labeled 'Revised Manuscript with Track Changes'.
- An unmarked version of your revised paper without tracked changes. You should upload this as a separate file labeled 'Manuscript'.

If you would like to make changes to your financial disclosure, please include your updated statement in your cover letter. Guidelines for resubmitting your figure files are available below the reviewer comments at the end of this letter.

If applicable, we recommend that you deposit your laboratory protocols in protocols.io to enhance the reproducibility of your results. Protocols.io assigns your protocol its own identifier (DOI) so that it can be cited independently in the future. For instructions see: https://journals.plos.org/plosone/s/submission-guidelines#loc-laboratory-protocols. Additionally, PLOS One offers an option for publishing peer-reviewed Lab Protocol articles, which describe protocols hosted on protocols.io. Read more information on sharing protocols at https://plos.org/protocols?utm_medium=editorial-email&utm_source=authorletters&utm_campaign=protocols.

As the corresponding author, your ORCID iD is verified in the submission system and will appear in the published article. PLOS supports the use of ORCID, and we encourage all coauthors to register for an ORCID iD and use it as well. Please encourage your coauthors to verify their ORCID iD within the submission system before final acceptance, as unverified ORCID iDs will not appear in the published article. *Only*  the individual author can complete the verification step; PLOS staff *cannot*  verify ORCID iDs on behalf of authors.

We look forward to receiving your revised manuscript.

Kind regards,

Dalton Müller Pessôa Filho, Ph.D.

Academic Editor

PLOS One

Journal Requirements:

If the reviewer comments include a recommendation to cite specific previously published works, please review and evaluate these publications to determine whether they are relevant and should be cited. There is no requirement to cite these works unless the editor has indicated otherwise.

Please review your reference list to ensure that it is complete and correct. If you have cited papers that have been retracted, please include the rationale for doing so in the manuscript text, or remove these references and replace them with relevant current references. Any changes to the reference list should be mentioned in the rebuttal letter that accompanies your revised manuscript. If you need to cite a retracted article, indicate the article’s retracted status in the References list and also include a citation and full reference for the retraction notice.

Additional Editor Comments:

Dear Authors,

First, please accept my sincere apologies for the delay in completing this second round of review. As you may know, securing reviewer feedback during the Northern Hemisphere summer vacation period can be particularly challenging, though some delays are naturally expected and understandable at this time of year.

I am pleased to share that the review process for round #2 is now complete. At this stage, only one reviewer has requested a few minor additional revisions to refine your manuscript further.

You will receive the formal decision letter along with the detailed reviewer comments shortly through the journal system. We look forward to receiving your revised manuscript and moving toward a final decision.

Thank you for your patience and for submitting your work to PLOS ONE.

Kind regards,

[Note: HTML markup is below. Please do not edit.]

Reviewers' comments:

Reviewer's Responses to Questions

**Comments to the Author**

Reviewer #1: (No Response)

Reviewer #2: All comments have been addressed

**********

2. Is the manuscript technically sound, and do the data support the conclusions?

Reviewer #1: Yes

Reviewer #2: Yes

**********

3. Has the statistical analysis been performed appropriately and rigorously?

Reviewer #1: Yes

Reviewer #2: Yes

**********

4. Have the authors made all data underlying the findings in their manuscript fully available?

The PLOS Data policy

Reviewer #1: No

Reviewer #2: Yes

**********

5. Is the manuscript presented in an intelligible fashion and written in standard English?

Reviewer #1: Yes

Reviewer #2: Yes

**********

Reviewer #1: Thank you for the detailed responses and the revisions made to the manuscript. The paper has clearly improved, especially regarding the clarification of its novelty, the methodological explanations, the more cautious interpretation of the results, and the addition of a limitations section.

I only have a few minor comments before publication:

1. The explanation about the pairwise comparisons could be expanded slightly. Some non-significant differences between Olympic Games separated by several decades still seem unexpected. A much better explanation of how the variability in the data and the multiple-comparison procedure may have influenced these results would help readers understand these findings.

2. The presentation of the percentage changes between consecutive Olympic Games should also be improved. Since the tables already include the mean performance values for each Olympic edition, I suggest adding one extra column showing the percentage change compared with the previous Olympic Games (or providing these data as supplementary material if space is an issue). This would make the evolution of performance much easier to follow without requiring any additional analyses.

Overall, I believe the authors have addressed the main concerns raised during the first review, and I consider the manuscript suitable for publication after these minor revisions.

Reviewer #2: A very significant work for the science and practice of top-level sports and swimming. Such research represents the foundation for summarizing knowledge about the development of a sport.

**********

what does this mean?). If published, this will include your full peer review and any attached files.

If you choose “no”, your identity will remain anonymous but your review may still be made public.

**Do you want your identity to be public for this peer review?**  For information about this choice, including consent withdrawal, please see our Privacy Policy

Reviewer #1: No

Reviewer #2: **Yes:**  Milivoj Dopsaj

**********

[NOTE: If reviewer comments were submitted as an attachment file, they will be attached to this email and accessible via the submission site. Please log into your account, locate the manuscript record, and check for the action link "View Attachments". If this link does not appear, there are no attachment files.]

To ensure your figures meet our technical requirements, please review our figure guidelines: https://journals.plos.org/plosone/s/figures

You may also use PLOS’s free figure tool, NAAS, to help you prepare publication quality figures: https://journals.plos.org/plosone/s/figures#loc-tools-for-figure-preparation.

NAAS will assess whether your figures meet our technical requirements by comparing each figure against our figure specifications.

Attachment Submitted filename: RW II MD_corections_list_25082026.docx

# Author comment: Author response to Decision Letter 2

**DOI:** 10.1371/journal.pone.0361289.r004

2 Oct 2026

Dear Editor and Reviewers,

We sincerely thank the Editor and the Reviewers for their careful evaluation of our manuscript and for their constructive comments and suggestions. We have carefully addressed all comments raised during this revision round and revised the manuscript accordingly.

In response to Reviewer #1, we expanded the Discussion to provide further clarification regarding the interpretation of pairwise comparisons, particularly the influence of within-edition variability and multiple-comparison procedures. We also clarified in the Data Analysis section that post hoc pairwise comparisons were performed using Holm–Sidak, Tukey, or Dunn’s procedures. In addition, Supplementary Tables S1–S9 have been provided to present the percentage changes between consecutive available Olympic Games for final performance times, swimming speeds, and reaction times, as applicable.

We sincerely thank Reviewer #2 for the positive assessment of our work. We have also addressed the additional technical corrections indicated by the reviewer, including the typographical correction in Table 3 and the requested formatting corrections in Tables 3, 4, 5, and 9.

A detailed point-by-point response to all reviewer comments has been provided in the separate “Response to Reviewers” file. All corresponding changes are shown in the marked-up manuscript.

Thank you for your consideration of our revised manuscript.

Attachment Submitted filename: responses to reviewers.docx

# Peer review: Decision Letter 2

**DOI:** 10.1371/journal.pone.0361289.r005

**Authors:** Dalton Pessôa Filho

9 Oct 2026

Performance Evolution in Olympic Swimming Over the Last 100 Years: A Retrospective Stroke-Based Analysis

PONE-D-26-00592R2

Dear Dr. Cengizel,

We’re pleased to inform you that your manuscript has been judged scientifically suitable for publication and will be formally accepted for publication once it meets all outstanding technical requirements.

Within one week, you’ll receive an e-mail detailing the required amendments. When these have been addressed, you’ll receive a formal acceptance letter and your manuscript will be scheduled for publication.

An invoice will be generated when your article is formally accepted. Please note, if your institution has a publishing partnership with PLOS and your article meets the relevant criteria, all or part of your publication costs will be covered. Please make sure your user information is up-to-date by logging into Editorial Manager at Editorial Manager® and clicking the ‘Update My Information' link at the top of the page. For questions related to billing, please contact billing support.

If your institution or institutions have a press office, please notify them about your upcoming paper to help maximize its impact. If they’ll be preparing press materials, please inform our press team as soon as possible -- no later than 48 hours after receiving the formal acceptance. Your manuscript will remain under strict press embargo until 2 pm Eastern Time on the date of publication. For more information, please contact onepress@plos.org.

Kind regards,

Dalton Müller Pessôa Filho, Ph.D.

Academic Editor

PLOS One

Additional Editor Comments (optional):

Dear Authors,

Thank you for your detailed response and for the significant effort put into revising the manuscript.

The reviewers have no further questions or comments.

Congratulations on a much-improved manuscript.

Reviewers' comments:

Reviewer's Responses to Questions

**Comments to the Author**

Reviewer #1: All comments have been addressed

**********

2. Is the manuscript technically sound, and do the data support the conclusions?

Reviewer #1: Yes

**********

3. Has the statistical analysis been performed appropriately and rigorously?

Reviewer #1: Yes

**********

4. Have the authors made all data underlying the findings in their manuscript fully available?

The PLOS Data policy

Reviewer #1: Yes

**********

5. Is the manuscript presented in an intelligible fashion and written in standard English?

Reviewer #1: Yes

**********

Reviewer #1: I would like to congratulate the authors for the excellent work. The manuscript has been greatly improved throughout the revision process, and the authors have addressed the reviewers’ comments very carefully. Congratulations.

**********

what does this mean?). If published, this will include your full peer review and any attached files.

If you choose “no”, your identity will remain anonymous but your review may still be made public.

**Do you want your identity to be public for this peer review?**  For information about this choice, including consent withdrawal, please see our Privacy Policy

Reviewer #1: **Yes:**  Tiago A. F. Almeida

**********

# Editor report: Acceptance letter

**DOI:** 10.1371/journal.pone.0361289.r006

**Authors:** Dalton Pessôa Filho

PONE-D-26-00592R2

PLOS One

Dear Dr. Cengizel,

I'm pleased to inform you that your manuscript has been deemed suitable for publication in PLOS One. Congratulations! Your manuscript is now being handed over to our production team.

At this stage, our production department will prepare your paper for publication. This includes ensuring the following:

* All references, tables, and figures are properly cited

* All relevant supporting information is included in the manuscript submission,

* There are no issues that prevent the paper from being properly typeset

You will receive further instructions from the production team, including instructions on how to review your proof when it is ready. Please keep in mind that we are working through a large volume of accepted articles, so please give us a few days to review your paper and let you know the next and final steps.

Lastly, if your institution or institutions have a press office, please let them know about your upcoming paper now to help maximize its impact. If they'll be preparing press materials, please inform our press team within the next 48 hours. Your manuscript will remain under strict press embargo until 2 pm Eastern Time on the date of publication. For more information, please contact onepress@plos.org.

You will receive an invoice from PLOS for your publication fee after your manuscript has reached the completed accept phase. If you receive an email requesting payment before acceptance or for any other service, this may be a phishing scheme. Learn how to identify phishing emails and protect your accounts at https://explore.plos.org/phishing.

If we can help with anything else, please email us at customercare@plos.org.

Thank you for submitting your work to PLOS One and supporting open access.

Kind regards,

PLOS One Editorial Office Staff

on behalf of

Prof. Dr. Dalton Müller Pessôa Filho

Academic Editor

PLOS One
